# A Phase I Randomized Clinical Trial of Candidate Human Immunodeficiency Virus type 1 Vaccine MVA.HIVA Administered to Gambian Infants

**DOI:** 10.1371/journal.pone.0078289

**Published:** 2013-10-24

**Authors:** Muhammed O. Afolabi, Jorjoh Ndure, Abdoulie Drammeh, Fatoumatta Darboe, Shams-Rony Mehedi, Sarah L. Rowland-Jones, Nicola Borthwick, Antony Black, Gwen Ambler, Grace C. John-Stewart, Marie Reilly, Tomáš Hanke, Katie L. Flanagan

**Affiliations:** 1 Vaccinology Theme, Medical Research Council Unit, Fajara, The Gambia; 2 Statistics and Data Management Department, Medical Research Council Unit, Fajara, The Gambia; 3 The Jenner Institute, University of Oxford, Oxford, United Kingdom; 4 Departments of Biostatistics, Medicine, and Epidemiology, University of Washington, Seattle, Washington, United States of America; 5 Department of Medical Epidemiology and Biostatistics, Stockholm, Sweden; Menzies School of Health Research, Australia

## Abstract

**Background:**

A vaccine to decrease transmission of human immunodeficiency virus type 1 (HIV-1) during breast-feeding would complement efforts to eliminate infant HIV-1 infection by antiretroviral therapy. Relative to adults, infants have distinct immune development, potentially high-risk of transmission when exposed to HIV-1 and rapid progression to AIDS when infected. To date, there have been only three published HIV-1 vaccine trials in infants.

**Trial Design:**

We conducted a randomized phase I clinical trial PedVacc 001 assessing the feasibility, safety and immunogenicity of a single dose of candidate vaccine MVA.HIVA administered intramuscularly to 20-week-old infants born to HIV-1-negative mothers in The Gambia.

**Methods:**

Infants were followed to 9 months of age with assessment of safety, immunogenicity and interference with Expanded Program on Immunization (EPI) vaccines. The trial is the first stage of developing more complex prime-boost vaccination strategies against breast milk transmission of HIV-1.

**Results:**

From March to October 2010, 48 infants (24 vaccine and 24 no-treatment) were enrolled with 100% retention. The MVA.HIVA vaccine was safe with no difference in adverse events between vaccinees and untreated infants. Two vaccine recipients (9%) and no controls had positive *ex* *vivo* interferon-γ ELISPOT assay responses. Antibody levels elicited to the EPI vaccines, which included diphtheria, tetanus, whole-cell pertussis, hepatitis B virus, *Haemophilus influenzae* type b and oral poliovirus, reached protective levels for the vast majority and were similar between the two arms.

**Conclusions:**

A single low-dose of MVA.HIVA administered to 20-week-old infants in The Gambia was found to be safe and without interference with the induction of protective antibody levels by EPI vaccines, but did not alone induce sufficient HIV-1-specific responses. These data support the use of MVA carrying other transgenes as a boosting vector within more complex prime-boost vaccine strategies against transmission of HIV-1 and/or other infections in this age group.

**Trial Registration:**

ClinicalTrials.gov NCT00982579

The Pan African Clinical Trials Registry PACTR2008120000904116

## Introduction

An unacceptably high number of children become infected with the human immunodeficiency virus type 1 (HIV-1) every year [1]. The majority of children who become infected with HIV-1 acquire the virus from their infected mothers during pregnancy, labour, delivery or breast-feeding. Although approximately 42% of mother-to-child transmission (MTCT) is due to prolonged breast-feeding [2-4], for many HIV-1-positive mothers formula feeding is not an option for social, practical and health reasons; breast-feeding reduces infant mortality due to nutrition and protection against other common childhood diseases [5]. Although antiretroviral therapy (ART) can significantly reduce the risk of MTCT, ARTs reach approximately 57% of HIV-1-infected mothers in low- and middle-income countries [6], and residual MTCT can occur despite ART [7]. Thus, development of safe, effective, accessible vaccines to decrease the prevalence of HIV-1 among mothers and to protect infants against their mother’s HIV-1 in the breast milk is a desired complement to the successful prevention of mother-to-child transmission of HIV-1 by ART and ultimately the best solution.

 Results of the first vaccine efficacy trials in adults suggested that an eventual successful anti-HIV-1 vaccine may need to induce both T cells and broadly neutralizing antibodies [8]. For protection against breast-milk HIV-1, immune responses must be elicited as early as possible after birth, and this will require priming at or soon after delivery followed by boost vaccination(s) within the first few months of life. 

 The natural history of HIV-1 infection and responsiveness to vaccinations may differ in adults and infants young/children [9,10], and parallel adult and paediatric clinical trials are required [11]. To date, there have been three published studies of active immunization in infants, which together evaluated 3 different Env-derived subunit proteins and 3 canarypox virus (ALVAC)-vectored vaccines. Thus, Pediatric AIDS Clinical Trials Group (PACTG) protocol 230 in the USA assessed safety and immunogenicity of Chiron gp120_SF-2_ adjuvanted with MF59 and VaxGen pg120_MN_ adsorbed into alum [12-14]. PACTG 326 carried out mainly in the USA conducted phase 1 testing of ALVAC vCP205 (expressing clade B GagProt_LAI_ and gp120_MN_), and in phase 2 ALVAC vCP1452 (expressing clade B GagProt_LAI_ plus several human T cell epitopes from Nef and Pol, and gp120_MN_ attached to the gp41_LAI_ transmembrane region) followed by a boost with AIDSVAX B/B [15] mixed gp120_MN_ and gp120_GNE8_ in alum; the first doses of rALVAC vaccines were delivered within 72 hours after birth [16,17]. Finally, the first African vaccine infant trial designated HIV Prevention Trials Network (HPTN) protocol 027 administered RV144 [18] ALVAC vCP1521 (expressing clade B GagProt_LAI_ and clade E gp120_92TH023_ linked to the transmembrane region of gp41_LAI_) to infants born to HIV-1-infected mothers in Uganda [19]. All vaccines in these three trials were safe and induced immune responses broadly similar to those observed in adults [20].

Modified vaccinia virus Ankara (MVA) is a highly attenuated non-replicating (in humans) poxvirus with an excellent safety and immunogenicity profile established in over 120,000 people vaccinated as a part of the smallpox eradication campaign [21] and its use as a vector for vaccines against a range of pathogens [22]. In the past, we designed and constructed immunogen HIVA (HIV clade A), which consists of consensus HIV-1 clade A Gag p24/p17 coupled to a string of partially overlapping CD8^+^ T-cell epitopes [23]. The HIVA vaccines were tested comprehensively pre-clinically including in non-human primates, whereby MVA.HIVA was less immunogenic in infants than in their mothers [24,25]. Delivered by DNA and MVA, HIVA was extensively tested in adults in the UK and Africa [26]. MVA.HIVA was shown to be a weak primer for the transgene product-specific CD4^+^ and CD8^+^ T-cells, but delivered a strong boost to well primed (e.g. by natural HIV-1 infection) responses [27-31]. With highly reassuring safety data from over 370 adult volunteers in the UK and Africa [27,30,32-35], we decided to start building foundations for testing vaccines against breast milk transmission of HIV-1 in two sub-Saharan African sites, The Gambia and Kenya, by using the ethics and regulatory review processes, establishing/expanding local infant vaccine trial capacity, and performing small vaccine trials PedVacc 001 and PedVacc 002 in infants born to HIV-1-uninfected and HIV-1-infected mothers. Here, we report on the PedVacc 001 trial, which administered a single low dose of MVA.HIVA to healthy 20-week-old infants born to HIV-1-uninfected mothers in The Gambia. This was the first time that a recombinant MVA vaccine with an HIV-1 transgene was administered to less than 1-year-old children in Africa.

## Methods

The protocol for this trial and supporting CONSORT checklist are available as supporting information; see Checklist S1 and Protocol S1.

### Study design

The Pediatric Vaccine (PedVacc) 001 study was a single-centre, phase I, open, randomized, no treatment-controlled study of candidate HIV-1 vaccine MVA.HIVA compared to no treatment control group. Randomization was generated by the Centre for Statistics in Medicine, University of Oxford using simple random sampling.

### Ethics and regulatory approvals

Approvals for the PedVacc 001 clinical study were granted by The Republic of The Gambia National Pharmaceutical Services Medicine Board, The Gambia Government/Medical Research Council (MRC) Joint Ethics Committee (ref. SC1106), Oxford Tropical Research Ethics Committee (OXTREC ref. 11 08) and The Stockholm Regional Ethics Committee (ref. 2009/1591-31/1). The study was conducted according to the principles of the Declaration of Helsinki (2008) and complied with the International Conference on Harmonization Good Clinical Practice guidelines. The trial was registered at the Clinical Trials.gov www.clinicaltrials.gov (ref. NCT00982579) and the Pan African Clinical Trials Registry www.pactr.org (ref. PACTR2008120000904116).

### Study population

The study was conducted at the MRC field site in Sukuta, a low-income peri-urban setting in the western region of The Gambia [36]. Mothers with infants born at Sukuta Health Centre were provided with study information and those interested in participating were invited to undergo voluntary HIV counseling and testing, since only infants of HIV-1/2-negative mothers were eligible. Infants underwent their routine Expanded Program on Immunization (EPI) vaccinations at 8, 12 and 16 weeks of age, namely Pentavalent vaccine (diphtheria, tetanus, whole cell pertussis (DTwP), hepatitis B virus (HBV) and *Haemophilus influenzae* type b (Hib)), oral poliovirus vaccine (OPV) and 7-valent pneumococcal conjugate vaccine (PCV-7) (all supplied by UNICEF, www.unicef.org) (Table 1). At 16 weeks of age, eligibility criteria were assessed and prospective written informed consent was obtained from all mothers of participating infants by signing and thumb-printing a filled form. Consent of fathers/partners was also sought, but was not a condition for participation. Eligible infants had to be healthy, afebrile, have no history of allergy or acute or chronic diseases, have a normal physical examination, and have received all EPI vaccines according to schedule. At a 19-week screening visit, 5 ml of blood was taken for baseline pre-vaccination immunogenicity, haematology and biochemistry analysis. At 20 weeks of age, the baseline blood tests were confirmed to be within range, eligible infants were randomized using sealed envelopes and assigned to vaccine (n=24) and no-treatment control (n=24) groups by a study nurse.

**Table 1:** EPI vaccination schedule in The Gambia.

**Age**	**Vaccine**
Birth	BCG, OPV, HBV
8 weeks	OPV, Pentavalent, PCV-7
12 weeks	OPV, Pentavalent, PCV-7
16 weeks	OPV, Pentavalent, PCV-7
9 months	Measles, Yellow fever, OPV

Pentavalent (DTwP, Hib, HBV), where DTwP - diphtheria, tetanus, whole cell pertussis; HBV - hepatitis B virus; Hib - *Haemophilus influenzae* type b; OPV - oral poliovirus vaccine; and PCV-7 – 7-valent pneumococcal conjugate vaccine

### The study vaccine and its administration

**Figure 1:**
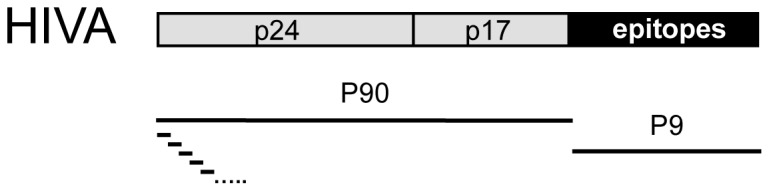
A schematic diagram of the HIV-1 clade A (HIVA) immunogen. The HIVA protein consists of consensus amino acid sequences of clade A p24 and p17 Gag and a string of partially overlapping CD8^+^ T-cell epitopes identified in chronically infected individuals [23]. Pool P90 of 15-mer peptides overlapping by 11 amino acids across the Gag portion is shown below the protein. Pool P9 consisted of known CD8^+^ T-cell epitope peptides derived from the polyepitope region.

MVA.HIVA is a recombinant non-replicating poxvirus, which carries the HIVA immunogen transgene (Figure 1) inserted by homologous recombination into the thymidine kinase locus of the parental MVA genome under the early/late P7.5 promoter [37]. MVA.HIVA was manufactured under current Good Manufacturing Practice conditions by Impfstoffwerk Dessau-Tornau (IDT), Germany. It was provided in vials of 200 μl at 5x10^8^ plaque-forming units (PFU)/ml in 10 mM Tris-HCl buffer pH 7.7 and 0.9% NaCl, and stored at -70°C. The appropriate number of vials were transported to the field site on dry ice on the day of administration, each vial was thawed at room temperature immediately prior to use and used within 1 hour of thawing. Participants in the vaccine group received a single intramuscular dose of 5x10⁷ PFU of MVA.HIVA considered to be a low adult dose, while the control group did not receive any treatment. The first vaccinated infant was observed for 24 hours to ensure there were no immediate safety concerns prior to vaccinating the remaining eligible participants. At subsequent visits at the age of 21, 28 and 36 weeks, a medical history was taken and a physical examination was performed.

### Blood sampling schedule and blood sample handling

Laboratory personnel were blinded to group allocation. Five ml of blood (4 ml EDTA and 1 ml clotted) was collected at 19, 21 and 28 weeks of age (i.e. pre-randomization, and 1 and 8 weeks post-randomization), and a 5-ml EDTA sample at 36 weeks (16 weeks post-randomization). MVA.HIVA immunogenicity was tested at all 4 time points; haematology and biochemistry at 19, 21 and 28 weeks; EPI vaccine antibody responses were determined at 19 and 21 weeks; and HIV testing was carried out at 28 weeks. Serum was collected from clotted blood. EDTA blood was used for full blood count (FBC) and HIV serology (week 28). Peripheral blood mononuclear cells (PBMC) were isolated from blood collected into EDTA by density gradient centrifugation on Lymphoprep cushion (Fresenius Kabi, Oslo, Norway).

### Safety monitoring

Vaccinated infants were monitored for 1 hour post-vaccination for immediate adverse events (AE), and by a home visit on each of the following two days to record AEs. At subsequent clinic visits, a medical history was taken, infants were examined and any AEs were documented. FBC, creatinine, alanine transaminase (ALT) and alkaline phosphatase (ALP) were tested at baseline (19 weeks) and at 1 week and 8 weeks post-vaccination. Local and systemic AEs and blood parameter abnormalities were graded from 0-3 as follows: absent (0), mild (1), moderate (2), severe (3). Relationship to MVA.HIVA vaccination was graded from 0-3 as follows: not related (0), possibly related (1), probably related (2), definitely related (3). Results were reviewed weekly by the trial safety monitor and externally monitored throughout the study. An external Data Monitoring and Ethics Committee reviewed safety data at 6 monthly intervals.

### Ex-vivo IFN-γ ELISPOT assay

Freshly isolated PBMC were suspended at 1x10^6^ cells/ml in R10 complete medium (RPMI1640 medium plus 10% FCS, L-glutamine, HEPES, sodium pyruvate, penicillin and streptomycin) and 200 μl (2x10^5^ cells) applied to each test well of 96-well plates (S5EJ044I10, Millipore). PBMC were stimulated in triplicate for reactivity to peptide pool 90 (P90) representing 88 peptides from the Gag p24/p17 and pool 9 (P9) containing 25 peptides covering the CD8^+^ T cell polyepitope regions of HIVA (Figure 1), both pools containing 1.5 μg/ml of each peptide. Phytohaemagglutinin (PHA) (10 mg/ml) and R10 alone were tested in triplicate wells as positive and background controls, respectively. ELISPOT plates were incubated at 37°C in 5% CO_2_ for 16 hours, and developed using the recommended protocol. Purple spots in each well, or spot-forming units (SFU), were read by the ELISPOT reader (Autoimmune Diagnostika software version 5.0, Strassburg, Germany). An assay failed quality control (QC) if the mean background was >20 SFU/well (>100 SFU/10^6^ PBMC), or mean PHA response was <30 SFU/well (<150 SFU/10^6^ PBMC). Poisson modeling was used to identify outliers among replicates, which were excluded, and Bonferroni correction was used for multiple testing. A response was considered positive if the mean stimulated response was at least twice the mean background response, and the ‘net response’ (background subtracted) was ≥ 50 SFU/10^6^ PBMC.

### EPI vaccine antibody responses

Microsphere-based multiplex assays were performed at the National Institute for Public Health and the Environment, Bilthoven, The Netherlands to quantify serum IgG antibodies against diphtheria toxin (Dtx), tetanus toxin (Ttx) and Hib as described previously [38]. Anti-HBV surface antigen (HBsAg) antibody levels were measured using an anti-HBsAg enzyme immunoassay kit (ETI-AB-AUK-3, Diasorin, Italy). Type 1 poliovirus IgG levels were determined by a neutralization assay as described previously [39].

### Anti-HIV-1 antibody testing

The Murex HIV-1 1.2.0 antibody assay kit (ABBOTT Murex, Dartford, UK) was used to test plasma samples for HIV-1 antibodies at 28 weeks of age.

### Statistical analysis

Data were analyzed using Stata version 12 (StataCorp, College Station, Texas) and Graphpad Prism software Version 6.0a (GraphPad Software Inc., California, USA). The immunology, vaccine antibody, haematology and biochemistry parameters were not normally distributed and non-parametric tests were used: two-tailed Mann-Whitney tests were used to compare variables between the vaccine and control groups and Wilcoxon matched-pairs tests for comparisons of responses for the same infants at different time points. A significance level of p<0.05 was considered statistically significant.

## Results

### Trial participants

**Figure 2:**
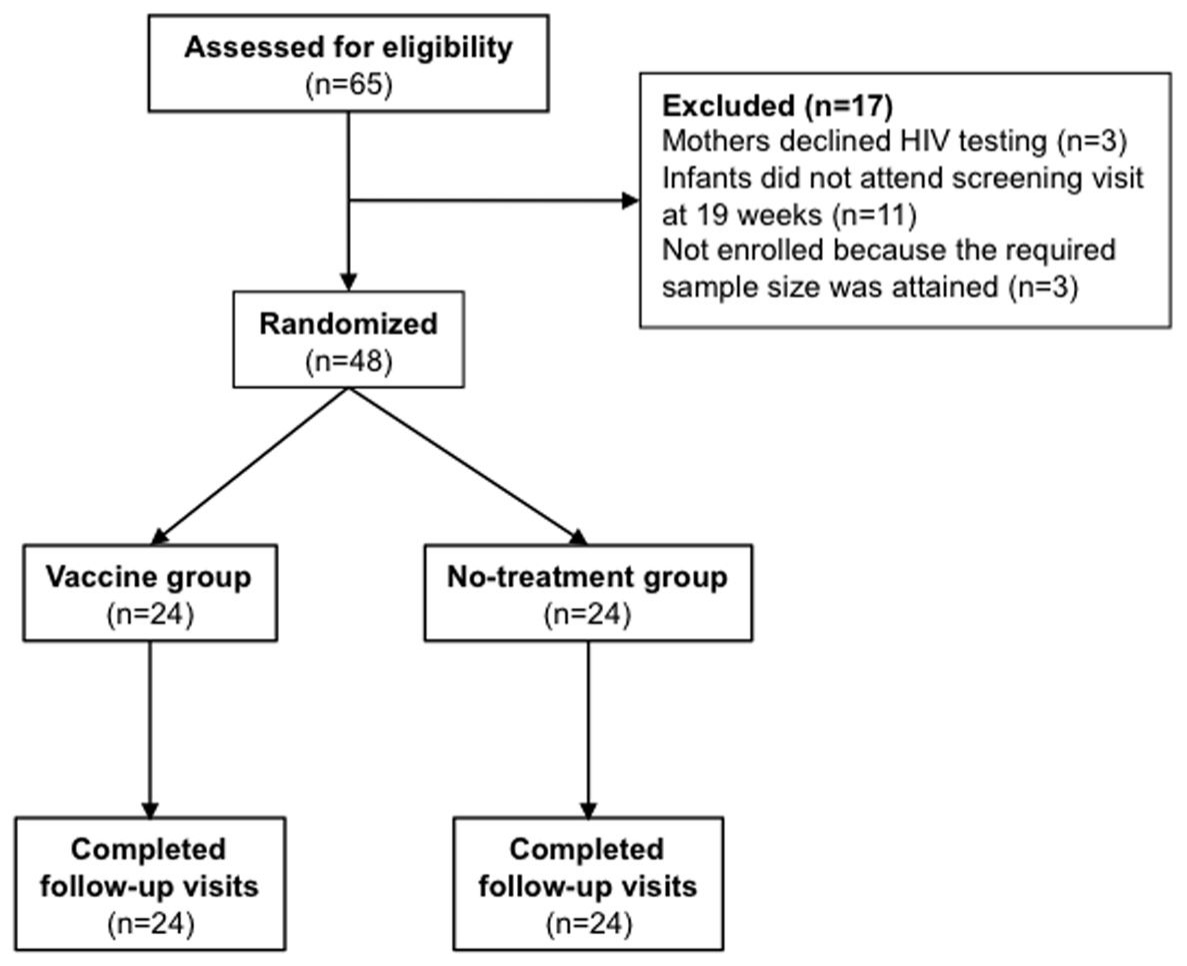
Trial Profile. Diagram indicating the numbers of infants screened and followed up throughout the study.

From March to October 2010, 64 infants were assessed for eligibility, of which 51 were screened at 19 weeks of age and 48 were randomized at 20 weeks to the vaccine (n = 24) or control (n = 24) groups (Figure 2). This was a pilot study with primarily descriptive outcomes. Groups were comparable by sex, age, weight and mid-upper-arm circumference (MUAC) on the day of vaccination at 20 weeks of age and also comparable at baseline (19 weeks) for WBC, creatinine, ALT and ALP (Table 2). However, baseline haemoglobin was higher in the vaccine group (11.3 g/dl vs. 10.8 g/dl; p=0.038). All 48 infants completed the study and all provided 4 blood samples.

**Table 2:** Baseline characteristics of study participants.

	**Control Group (n=24)**	**Vaccine Group (n=24)**	***p* value**
Sex	55% Male	67% Male	
	45% Female	33% Female	
Age (week)	20.0 (19.0, 20.0)	20.0 (19.8, 20.8)	0.280
Weight (kg)	7.2 (6.7, 7.7)	7.3 (6.9, 7.6)	0.680
MUAC (cm)	15.0 (14.4, 15.5)	15.0 (14.4, 15.4)	0.925
Hb (g/dl)	10.8 (10.2, 11.2)	11.3 (10.6, 11.9)	**0.038**
WBC (x10^9^ cell/l)	9.3 (7.9, 12.1)	9.1 (8.5, 11.8)	0.821
Platelets (x10^9^ cell/l)	493.0 (395.8, 637.5)	545.0 (414.8, 582.0)	0.915
Creatinine (mM)	24.5 (22.0, 31.2)	26.0 (23.0, 38.5)	0.860
ALT (U/l)	20.5 (16.8, 28.0)	21.0 (17.0, 25.5)	0.901
ALP (U/l)	293 (247, 344)	304 (249, 355)	0.749

Median values are shown with the interquartile range (IQR) in brackets. *p* values are shown for comparisons between the vaccine and control groups using a two-tailed Mann-Whitney U test. MUAC - mid upper arm circumference; Hb - haemaglobin; WBC - white blood cell count; ALT - alkaline transaminase; ALP - alkaline phosphatase.

### MVA.HIVA was well tolerated

There were no local reactions in the hour after MVA.HIVA vaccination, and only 1 infant developed mild redness (<50 mm), which persisted for 2 days, but resolved by day 7. No children showed signs of localized pain (Table 3).

**Table 3:** Local and systemic AEs in the first 7 days of follow up.

	**Vaccine Group (n=24)**			**Control Group (n=24)**		
	**Grade 1**	**Grade 2**	**Grade 3**	**Grade 1**	**Grade 2**	**Grade 3**
	**Mild**	**Moderate**	**Severe**	**Mild**	**Moderate**	**Severe**
Pain	0	0	0	NA	NA	NA
Redness	1 (4.2%)	0	0	NA	NA	NA
Induration	0	0	0	NA	NA	NA
Scaling	0	0	0	NA	NA	NA
Blistering	0	0	0	NA	NA	NA
Fever	3 (12.5%)	3 (12.5%)	0	0	3 (12.5%)	0
Excessive crying	1 (4.2%)	0	0	0	0	0
Poor appetite	0	0	0	0	0	0
Vomiting	2 (8.3%)	0	0	0	0	0
Diarrhoea	0	0	0	2 (8.3%)	0	0
Poor weight gain	7 (29.2%)	0	0	7 (29.2%)	0	0
Cough	4 (16.7%)	0	0	2 (8.3%)	0	0

Note some infants had more than 1 adverse event. The control group did not receive a vaccine hence no local reactogenicity data were recorded.

There were no serious AEs throughout the study. Five infants had mild AEs that were possibly vaccine related, consisting of low-grade fever (n = 2), vomiting (n = 2), and low-grade fever plus excessive crying (n = 1) (Table 3). A further 10 AEs in the vaccine group were considered unrelated to MVA.HIVA vaccination (9 mild, 1 moderate), including eye discharge (n = 2), rash (n = 1), and weight loss at 28 or 36 weeks (n = 7). The control group similarly had 3 cases of fever, 2 of rash and 7 cases of weight loss. One case of skin rash was of moderate severity in both the vaccine and control groups. There was no significant difference in weight or MUAC between the control and vaccine groups at any time point (not shown); and the 4 vaccinees and 5 control subjects who had weight loss at 28 weeks all returned to acceptable weight-for-age by 36 weeks of age.

There were no clinically significant biochemical or haematological abnormalities in vaccine recipients (Table 4). Haemoglobin levels were within range with the exception of 1 vaccinated and 1 control infant with mild low values, and 1 control infant with a high value. High WBC and platelet counts were observed at 21 and 28 weeks in both vaccinated and unvaccinated participants at baseline and after vaccination, although none were thought to be of clinical significance. The ALT levels remained in range for all but one vaccinated infant at baseline, and the creatinine level was low in up to one third of infants in both groups (lowest value 16 mM), which was not considered clinically significant. Alkaline phosphatase remained within range for all infants at all time points.

**Table 4:** Frequency of biochemical and haematological abnormalities.

		**Week 19**		**Week 21**		**Week 28**	
		**Vaccine**	**Control**	**Vaccine**	**Control**	**Vaccine**	**Control**
**Hb (g/dl)**	**High**	0	0	0	1 (4.3%)	0	0
**8-14**	**Low**	0	0	1 (4.3%)	0	0	1 (4.3%)
**WBC (cell/l)**	**High**	2 (9.1%)	0	5 (26.3%)	7 (41.2%)	4 (20.0%)	6 (33.3%)
**3.5-14x10^9^**	**Low**	0	0	0	0	0	0
**Platelets (cell/l)**	**High**	6 (37.5%)	9 (60%)	4 (21.1%)	5 (27.8%)	6 (33.3%)	6 (33.3%)
**150-600x10^9^**	**Low**	2 (12.5%)	0	1 (5.3%)	1 (5.6%)	0	0
**Creatinine (mM)**	**High**	0	0	0	0	0	0
**20-70**	**Low**	0	0	4 (20.0%)	2 (9.1%)	6 (33.3%)	5 (26.3%)
**ALT (U/l)**	**High**	1 (4.4%)	0	0	0	0	0
**10-80**	**Low**	0	0	0	0	1 (4.4%)	0
**ALP (U/l)**	**High**	0	0	0	0	0	0
**140-1000**	**Low**	0	0	0	0	0	0

The first column shows unit in brackets with acceptable ranges below. Numbers of infants with values above (high) and below (low) the normal range are indicated on the table with the percentage in brackets. Hb – hemoglobin; WBC – white blood cell count; ALT – alanine transaminase; ALP – alkaline phosphatase.

### Single low dose of MVA.HIVA did not induce sufficient anti-HIV-1 responses

**Figure 3:**
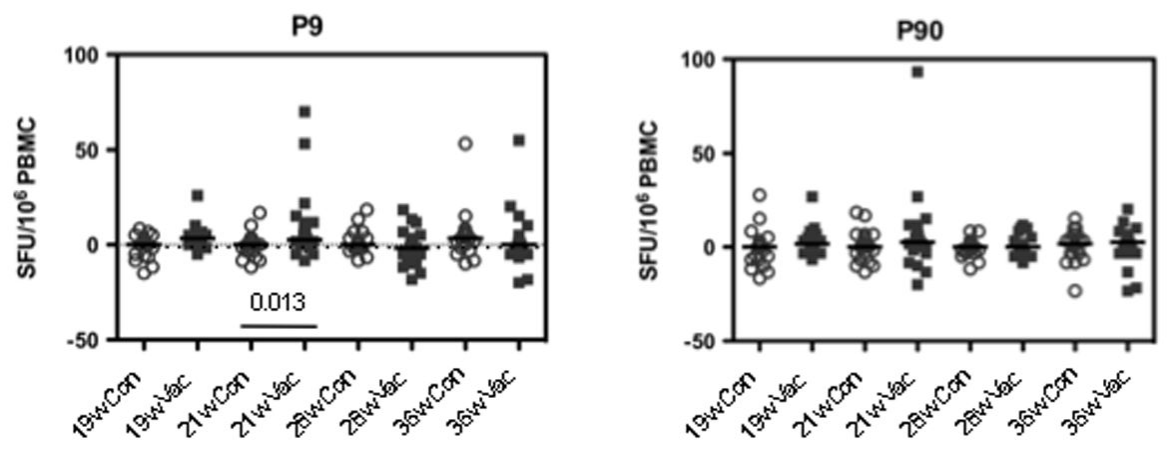
MVA.HIVA-elicited weak T-cell responses in fresh IFN-γ ELISPOT assay. The net fresh *ex-vivo* ELISPOT frequencies of IFN-γ-producing cells (mean of stimulated wells minus mean of negative control wells) to HIVA peptide pools P9 and P90 at all 4 bleed time points for the control (Con) and vaccinated (Vac) groups are shown. The p value for the only statistically significant difference between the two groups after Bonferroni correction is given. The median ‘mock’ no-peptide background response across all wells on plates that passed QC was 5 SFU/10^6^ PBMC (IQ range 0-20), and median PHA response was 1,850 SFU/10^6^ PBMC (IQ range 1,065- 2,960).

Immunogenicity of a single intramuscular low dose of MVA.HIVA was assessed using an *ex-vivo* IFN-γ ELISPOT assay. Altogether, 167 fresh PBMC samples of the total of 192 possible samples (48 infants x 4 time points) were assayed. Of these, 2 samples (1.2%) failed QC because of high background levels and none failed due to a low PHA response. None of the infants had HIV-1-specific T-cell responses before vaccination at 19 weeks of age. At 21 weeks, i.e. 1 week after the MVA.HIVA administration, 2 of 22 (9%) vaccine recipients had detectable HIV-1-specific, IFN-γ-producing T cells at net frequencies of 93 and 70 SFU/10^6^ PBMC, while no controls had detectable responses (Figure 3). There were no responders in either group at 28 weeks of age and at 36 weeks of age, one vaccinated and one unvaccinated infant had weakly-positive responses. Comparing the vaccine and no-treatment groups as a whole, only peptide pool P9-specific responses at week 21 were statistically higher in vaccine recipients (p = 0.013). 

### MVA.HIVA administration did not affect protective antibody levels induced by EPI vaccines

Antibody levels elicited by EPI vaccines were compared between MVA.HIVA vaccine recipients and untreated controls at 19 and 21 weeks, i.e. 1 week before and 1 week after the MVA.HIVA vaccination, respectively. This analysis showed that the two groups had comparable antibody responses to Ttx, Hib, HBV and OPV. All infants reached protective antibody levels except for 1 non-responder to HBV vaccine in the vaccine group at both 19 and 21 weeks, and 3 infants (2 vaccine recipients and 1 control) with Hib antibodies below protective levels at 21 weeks. Paired comparisons of antibody levels at 19 and 21 weeks showed that the control group, but not the vaccine group had significantly lower anti-HBsAg antibody levels at 21 weeks (p = 0.001) (Table 5). Dtx antibodies declined significantly in both control and vaccine groups (p = 0.043 and 0.0011), and Ttx antibodies decreased in the vaccine recipients only (p = 0.0053). Only the declines in Dtx and Ttx antibodies in the vaccine group were statistically significant after Bonferroni correction (p ≤ 0.0125), although they remained well above protective levels for all infants (Table 5).

**Table 5:** Tested antibody titres at 19 and 21 weeks of age elicited by EPI vaccines.

		**Control**		**p value (Control**	**Vaccine**		**p value (Vaccine**	**p value Con vs Vac**	
**EPI Vaccine**	**Protective Level**	**19 weeks**	**21 weeks**	**19 vs 21 weeks**)	**19 weeks**	**21 weeks**	**19 vs 21 weeks**)	**19 week**	**21 week**
**Poliovirus 1**	≥8	512	1024	0.313	512	512	0.629	0.765	0.616
**(neut. titre)**		(256, 1024)	(224, 1024)		(128, 1024)	(112, 1024)			
**HBsAg**	≥10	390	362	0.001*	358	311	0.098	0.349	0.570
**(IU/ml)**		(282, 537)	(212, 468)		(247, 418)	(232, 412)			
**Dtx**	≥0.01	1.39	1.19	**0.043**	1.73	1.46	0.0011*	**0.043**	0.105
**(IU/ml)**		(0.8, 1.8)	(0.8, 1.7)		(1.4, 2.2)	(1.0, 2.1)			
**Ttx**	≥0.01	3.71	3.39	0.422	3.03	2.54	0.0053*	0.762	0.376
**(IU/ml)**		(1.8, 5.6)	(1.4, 5.4)		(1.9, 4.5)	(1.5, 3.4)			
**Hib**	≥0.15	5.88	6.37	0.197	8.21	7.95	0.257	0.762	0.671
**(µg/ml)**		(2.1, 23.8)	(2.4, 24.8)		(3.0, 15.7)	(3.1, 17.6)			

Antibody responses to the EPI vaccines OPV (Poliovirus 1), HBV (HBsAg), diphtheria toxin (Dtx), tetaus toxin (Ttx) and Hib. Median values are shown with interquartile range in brackets below. Wilcoxon matched pairs analysis was used to compare 19 and 21 week responses for the same infants; 2-tailed Mann-Whitney *U* tests were used to compare vaccine and control groups. Significant p values are indicated in bold type, * indicates significance after Bonferroni correction. Vac - vaccine group; Con - control group.

### MVA.HIVA recipients remained HIV test negative

No vaccinated infant was found to be positive for HIV-1 or HIV-2 antibodies at 28 weeks of age, 8 weeks after the MVA.HIVA vaccine was administered. This concurs with previous results in adult trials [31].

## Discussion

The PedVacc 001 trial was conducted as a capacity building, feasibility and recombinant MVA safety study. The trial was approved by regulatory and all ethics bodies, and successfully recruited and retained 100% of infant-mother pairs. The MVA.HIVA vaccine was well tolerated and safe, but on its own, did not sufficient HIV-1-specific responses. PedVacc 001 is the first infant vaccine trial using recombinant MVA with an HIV-1-derived insert and only the second infant candidate HIV-1 vaccine trial conducted in sub-Saharan Africa. As such, this study contributed to the preparedness for conducting future infant HIV-1 vaccine trials in this region.

 In the PedVacc 001 trial, candidate HIV-1 vaccine MVA.HIVA demonstrated an excellent reactogenicity profile causing only a small number of mild AEs. There were no vaccine-related or other SAEs, no clinically significant haematological or biochemical disturbances and no significant adverse effects on infant growth; these results concur with studies of MVA-vectored vaccines for other diseases [40,41]. All out-of-range values for haematological and biochemical parameters were considered mild and clinically insignificant. The fact that WBC, platelet and creatinine values were frequently out of range in both vaccine recipients and controls suggests that locally appropriate ranges are wider than those adopted in this study. Indeed, there are no widely accepted normal values for infants in sub-Saharan Africa and this study contributes towards establishing such reference ranges [42-45].

 One low-dose administration of MVA.HIVA was marginally immunogenic by fresh IFN-γ ELISPOT assay frequencies, whereby only 9% of vaccinated infants developed detectable HIV-1-specific T-cell responses. Group analysis showed statistically significant responses to 1 of 2 tested peptide pools 1 week after MVA.HIVA administration, which concurs with the timing of peak responses observed in adult vaccinees [29]. Thus, MVA.HIVA administered alone was not sufficiently immunogenic. This was not totally unexpected. First, PedVacc 001 used a very prudent infant safety approach in administering a single low dose of the MVA.HIVA vaccine compared to 4 doses used in the other infant vaccine studies, which elicited lymphoproliferative, rare cytotoxic T-cell and Env-specific Ab responses [12-14,16,17,19]. Second, MVA-vectored vaccines are not strong primers of T-cell responses and as such are typically used for boosting in heterologous prime-boost regimens. In contrast after either a DNA prime or in HIV-1-infected volunteers on HAART, all MVA.HIVA recipients developed vaccine-stimulated responses [27-29,31,46-48]. In 2007 when the PedVacc 001 trial was conceived, we envisaged a strategy administering MVA.HIVA boost to 20-week-old infants who have been primed at birth with HIVA-expressing *Mycobacterium bovis* bacillus Calmette-Guérin (BCG) [49,50]. Moreover, encouraged by then promising results of candidate TB vaccine MVA85A [51], there was a possibility to develop the BGC-MVA regimen into a dual HIV-TB vaccine platform [24,25,52-56]. Since the commencement of PedVacc 001, both the immunogen design and vector delivery evolved. Thus, we showed that a prime with non-replicating recombinant adenovirus of chimpanzee origin followed by a boost with recombinant MVA induced uniquely high frequencies of HIV-1-specific T cells in UK adults [57]. As for the immunogen, HIVA has been superseded by universal pan-clade immunogen HIVconsv, which is based on the 14 most conserved regions of the HIV-1 proteome to tackle HIV-1 diversity and escape [57-59]. Furthermore, we plan to include component(s) inducing broadly neutralizing antibodies into the final vaccine regimen when these become available. Thus, although further clinical development of the MVA.HIVA vaccine has now ceased, safety demonstrated in PedVacc 001 builds confidence for future use of the MVA vector in heterologous vaccine strategies in this age group in Africa.

A single MVA.HIVA dose administered 4 weeks after the busy EPI vaccine period did not significantly affect the immunogenicity of EPI vaccines, nor influenced the number of infants with vaccine antibody titers above protective levels. This agrees with the results from 3 previous HIV-1-vaccine studies [13,16,19]. Notably, a reduction of MVA85A immunogenicity in Gambian infants was reported when co-administered with EPI [40].

Overall, this small phase I trial demonstrates that challenges associated with studies in infants such as obtaining ethical and regulatory approvals, logistics of recruiting and retaining mother-infant pairs and working with small blood volumes for immune assays can be overcome. As part of the trial conduct, infrastructure improvements significantly increased the capacity and daily efficiency of the health centre in Sukuta, including provision of laboratory, GCP, data and project management training. Thus, PedVacc 001 represents another cumulative step towards developing a prime-boost vaccine regimen aimed at reducing the transmission of HIV-1 during breast-feeding.

## Supporting Information

Checklist S1 **CONSORT 2010 checklist of information on the clinical trial PedVac 001 as presented in this manuscript.**
(PDF)

Protocol S1 **PedVac 001 clinical trial protocol.**
(PDF)
