# Supplementary material for: A Phase I Randomized Clinical Trial of Candidate Human Immunodeficiency Virus type 1 Vaccine MVA.HIVA Administered to Gambian Infants
Source: PLoS One. 2013 Oct 24;8(10):e78289. doi: 10.1371/journal.pone.0078289 (PMC3813444; doi:10.1371/journal.pone.0078289)
Supplement: Protocol S1 — PedVac 001 clinical trial protocol. (PDF) [file pone.0078289.s002.pdf]

Title: **AN OPEN RANDOMIZED PHASE I STUDY EVALUATING SAFETY AND IMMUNOGENICITY OF A CANDIDATE HIV-1 VACCINE, MVA.HIVA, ADMINISTERED TO HEALTHY INFANTS BORN TO HIV-1/2-UNINFECTED MOTHERS**

PV001 CLINICAL TRIAL PROTOCOL

Medical Research Council Laboratories, Fajara
Scientific Coordinating Committee Study No: SCC1106
OXTREC Reference No: 11 08

Sponsor: Medical Research Council, UK

Principal Investigator: Dr Katie Flanagan

Local Safety Monitor: Dr Uduak Okomo

Version Number: 8.0
7th May 2010

SIGNATURE PAGE

The signature below constitutes the approval of this protocol and the attachments, and provides the necessary assurances that this trial will be conducted according to all stipulations of the protocol, including all statements regarding confidentiality, and according to local legal and regulatory requirements and applicable UK regulations and ICH guidelines.

Chief Investigator:

Signed: [signature] Date: June 7, 2010

Tomas Hanke, BSc, MSc, PhD

Principal Investigator:

Signed: [signature] Date: 8/6/10.

Katie Flanagan, BA (Hons), MBBS, FRCP, DTM&H, PhD, CCST
Infant Immunologist

Sponsor:

Signed: [signature] Date: 7/6/10

Medical Research Council, UK

CONFIDENTIAL

TABLE OF CONTENTS

		Page
**1**	**KEY ROLES**	**8**
**2**	**BACKGROUND INFORMATION AND SCIENTIFIC RATIONALE**	**8**
**2.1**	**The need for an HIV vaccine against mother-to-child transmission**	**9**
**2.2**	**The investigational MVA.HIVA Vaccine**	**10**
2.2.1	MVA.HIVA design	10
2.2.2	Pre-clinical studies with MVA-vectored recombinant vaccines	11
2.2.3	Pre-clinical safety and immunogenicity of MVA.HIVA	11
2.2.4	Safety of recombinant MVA vaccines in humans	11
2.2.5	Safety of MVA.HIVA in adult humans.	12
2.2.6	Immunogenicity of MVA.HIVA in clinical trials	13
2.2.7	Containment and transmission of live MVA-vectored vaccines	13
**2.3**	**Potential Risks and Benefits**	**14**
2.3.1	Potential Risks	14
2.3.2	Known Potential Benefits	14
**3**	**OBJECTIVES**	**15**
**4**	**STUDY DESIGN**	**15**
**4.1**	**Sample size:**	**16**
**4.2**	**Randomisation**	**17**
**5**	**STUDY POPULATION**	**17**
**5.1**	**Inclusion Criteria**	**17**
**5.2**	**Exclusion Criteria**	**18**
**5.3**	**Sensitisation and Recruitment Procedures**	**18**
**6**	**STUDY ENROLMENT AND SCHEDULE OF FOLLOW-UP VISITS**	**19**
**6.1**	**Laboratory Tests**	**20**
**6.2**	**Study Visits**	**20**
**7**	**STUDY INTERVENTION / INVESTIGATIONAL PRODUCT**	**24**
**8**	**ASSESSMENT OF SCIENTIFIC OBJECTIVES**	**24**
**9**	**ASSESSMENT OF SAFETY**	**25**

9.1 Definitions 25

9.2 Adverse Events Assessment 26

9.3 Adverse Event Monitoring 28

10 CLINICAL MONITORING STRUCTURE 29

11 STATISTICAL CONSIDERATIONS 31

11.1 Determination of the Sample Size 31

11.2 Expected analyses and outcomes 31

11.2.1 Safety of MVA.HIVA 31

11.2.2 Immunogenicity of MVA.HIVA 31

11.2.3 Immunogenicity of EPI vaccines 31

11.3 Data Management 31

12 SOURCE DOCUMENTS AND ACCESS TO SOURCE DATA/DOCUMENTS 32

13 QUALITY CONTROL AND QUALITY ASSURANCE 32

14 ETHICS/PROTECTION OF HUMAN SUBJECTS 32

14.1 Informed Consent Process 33

14.2 Subject Confidentiality 33

14.3 Biohazard Containment 33

15 DATA HANDLING AND RECORD KEEPING 33

16 PLANS FOR DISTRIBUTION OF RESEARCH FINDINGS TO STUDY COMMUNITY 35

17 REFERENCES 35

APPENDIX 1 - EQUIPMENT FOR FIELDWORK 39

LIST OF ABBREVIATIONS

**AIDS**	Acquired immune deficiency syndrome
**AE**	Adverse event
**ALT**	Alanine aminotransferase
**ANC**	Antenatal clinic
**CRF**	Case Report Form
**CRN**	Clinical Research Nurse
**CTL**	Cytotoxic T lymphocyte
**DMEC**	Data Monitoring & Ethics Committee
**DTwP**	Diphtheria, tetanus, whole cell pertussis combined vaccine
**ELISA**	Enzyme-linked immunosorbant assay
**ELISPOT**	Enzyme-linked immunospot
**EPI**	Expanded Progamme of Immunization
**FBC**	Full blood count
**GCP**	Good Clinical Practice
**GTAC**	Gene Therapy Advisory Committee
**HepB**	Hepatitis B virus vaccine
**Hib**	*Haemophilus influenzae* b vaccine
**HIV**	Human immunodeficiency virus
**HLA**	Human leukocyte antigen
**ICH**	International Conference on Harmonization
**IFN-γ**	Gamma interferon
**LFT**	Liver function test
**LSM**	Local safety monitor
**MHRA**	Medicines and Healthcare Products Regulatory Agency
**MUAC**	Mid left upper arm circumference
**MVA.HIVA**	Recombinant non-replicating modified vaccinia virus Ankara expressing HIV-derived immunogen HIVA
**NAb**	Neutralizing antibodies
**NHS**	National Health Service
**OPV**	Oral poliovirus vaccine
**PBMC**	Peripheral blood mononuclear cells
**PCR**	Polymerase chain reaction
**PCV-7**	7-valent polysaccharide conjugate pneumococcal vaccine
**Pentavalent**	Pentavalent vaccine containing DTwP, Hib, HepB

**SAE**	Serious adverse event
**SoAE**	Solicited adverse event
**SUSAR**	Suspected unexpected serious adverse reactions
**TSC**	Trial Steering Committee
**VCT**	Voluntary counselling and testing

PROTOCOL SUMMARY

Title:
An open randomized phase I study evaluating safety and immunogenicity of a candidate HIV-1 vaccine, MVA.HIVA, administered to healthy infants born to HIV-1/2-uninfected mothers.

Phase: Phase I

Population:
N = 48: (2 x 24) = 48 healthy infants who have received all EPI vaccines to date

Number of Sites:
One: Sukuta Health Centre, The Gambia

Study Duration:
18 months

Description of Investigational Product/ Intervention:
1 dose of 5×10^7 pfu of MVA.HIVA administered intramuscularly

Blinding: There will be no blinding in the field, but the samples will be coded and the randomisation group will not be known to the laboratory staff performing and analysing the immunological assays.

Objectives:
Primary: Safety and immunogenicity of MVA.HIVA vaccine in 20-week-old healthy Gambian infants born to HIV-1/2-uninfected mothers.

Secondary: Gross impact of MVA.HIVA on the immunogenicity of EPI vaccines (DTwPHib, HepB, PCV-7 and OPV) when administered at 20 weeks (4 weeks after the last EPI vaccines), who have had BCG vaccine within the first 4 weeks of life.

Description of Study Design:
This is a randomised phase I study of a candidate HIV vaccine, MVA.HIVA, in healthy infants born to HIV-1/2-uninfected mothers. The immunology lab will be blinded for the investigational product allocation.

CONFIDENTIAL

1 KEY ROLES

For questions regarding this protocol, contact:

Dr Katie Flanagan, BA Hons, MBBS, FRCP, DTM&H, PhD, CCST, Principal Investigator
Postdoctoral Immunologist
Medical Research Council Laboratories
P. O. Box 273, Banjul, The Gambia
Phone +220 4496647 Email: kflanagan@mrc.gm

Dr Tomas Hanke, BSc, MSc, PhD, Chief Investigator
Weatherall Institute of Molecular Medicine
The John Radcliffe
University of Oxford
Oxford OX3 9DS
Phone 01865 222355 Email: tomas.hanke@imm.ox.ac.uk

Institutions:

Sponsor:	Medical Research Council, 20 Park Crescent, London W1B 1AL
External Monitor:	Appledown Clinical Research Ltd, Orchard's End, Greenland's Lane, Prestwood, Great Missenden, Bucks, HP16 9QX
Study sites:	Sukuta Health Centre, Fajara, The Gambia
Clinical laboratory:	MRC Laboratories, Fajara, The Gambia

Investigators:

Katie Flanagan	MRC	Principal Investigator / Infant Immunologist
Martin Ota	MRC	Clinical Immunologist / Vaccinologist
Muhammed Afolabi	MRC	Research Clinician
Jorjoh Ndure	MRC	Scientific Officer
David Jeffries	MRC	Statistician
Mehedi Shams-Rony	MRC	Database Manager
Yam Ndow Jallow	DoSH	Gambia Government Acting Head of EPI
Hilton Whittle	MRC	Emeritus Scientist
Sarah Rowland-Jones	MRC	MRC Professor
Tomas Hanke	U of Oxford	Reader

Local Officials

Vivat Thomas	MRC	Local Trial Monitor
Dr Uduak Okomo	MRC	Local Safety Monitor
Jenny Mueller	MRC	Clinical Quality Assurance Officer

2 BACKGROUND INFORMATION AND SCIENTIFIC RATIONALE

2.1 The need for an HIV vaccine against mother-to-child transmission

Since the first report of AIDS in 1981, it is estimated that over 60 million people have become infected with HIV-1, of whom some 25 million have died. Over 60% of the global HIV-1-infected population lives in Africa and about half of the infected adults are women of child-bearing age. Up to 40% of pregnant women attending larger urban centre clinics in Kenya are HIV-1 seropositive. Despite the fact that approximately half of mother-to-child transmission (MTCT) is due to prolonged breast-feeding [1], for many HIV-1-infected mothers bottle-feeding is not an option for social, practical and health reasons. Bottle-fed babies often have a higher morbidity and mortality due to increased exposure and susceptibility to other infections [2]. Although antiretroviral therapy can significantly reduce the risk of MTCT at parturition, it is less clear whether it is practical to use these drugs to prevent HIV-1 transmission by breast milk. The drugs are expensive, would have to be administered at birth and treatment has to be maintained throughout the whole period of breastfeeding; furthermore their effectiveness might be compromised by emergence of resistant mutants. Thus, one of the best hopes for protecting newborns and infants in developing countries against MTCT of HIV-1 is through a safe, effective, accessible prophylactic vaccine, which would both reduce the adult burden of infection and/or protect neonates against vertical HIV-1 transmission.

There is no doubt that a vaccine inducing both neutralizing antibodies and T cells will have the best chance to benefit vaccinees. However, development of vaccines inducing broadly neutralizing antibodies against HIV-1 has proven impossible so far and is clearly extremely difficult to achieve. The alternative approach exemplified by many HIV-1 vaccines currently in trials focuses on the induction of T cell immunity. T cells function by killing HIV-1-infected cells and producing soluble factors that can directly and indirectly control HIV-1 spread. While T cells cannot prevent transmitted virus from infecting host cells, they could limit the extent of early viral infection, and are expected to increase the dose of incoming HIV-1 necessary to establish infection. As a result, potent vaccine-induced HIV-1-specific T cell responses could decrease tissue damage during the acute phase of infection and improve the control of the virus load leading to a lower set point, thus delaying the development of AIDS and reducing viral transmission. The current T cell vaccination protocols can achieve a partial protection in non-human primate challenge models, in which animals are infected with higher doses of and more pathogenic viruses than those encountered by humans. Therefore there is a good case for believing that induction of T cell responses by vaccination will translate into a real benefit for human recipients. In animal models, qualitative differences in memory T cells may dramatically influence vaccine-mediated protective immunity even when the frequencies of HIV-1-specific T cells are similar [3]. Thus, to test the T cell hypothesis adequately, means of inducing high frequencies of multifunctional, durable T cells with strong proliferative potential and recognizing protective rather than immunodominant epitopes, will need to be developed. Recent discontinuation of vaccination and enrolment in phase IIb trials of Merck's human adenovirus serotype 5 (MRK rAd5)-vectored vaccine should be noted, but it is not a reason for general dismissal of the T cell strategy. Both the Merck vaccine delivery vector and HIV-1-derived immunogens can be improved on and thus the results of these trials are regarded as a 'product' rather than 'concept failure'. Clearly, more vaccine concepts need to be tested in phase IIb test-of-principle trials. Furthermore, once a Nab-

inducing vaccine becomes available, this can be combined with the best performing T cell vaccine for maximum benefit.

It is not known how early in life T cells can be educated to launch a protective response against intracellular microorganisms and this most likely differs from pathogen to pathogen. Qualitative and quantitative differences between responses in human newborns and adults are observed for a number of infections [4-7]. In HIV-1-infected infants, lower CD8 T cell responses compared to adults may play an important role in the faster disease progression [8-10]; children account for 4% of HIV-1 infections, yet they represent 20% of AIDS deaths [5]. At the same time, mature responses to certain infections and vaccines have been demonstrated during the postnatal and even foetal period [11]. This is particularly true for BCG vaccine-induced responses, which promote adult-like Th1 response in newborns [12-15]. Therefore, there is some evidence suggesting that protective T cell-mediated responses could be elicited by vaccines in early life, and BCG as a vaccine vector might be very well suited to prime them [16].

Taken together, it is our hypothesis that priming HIV-1-specific responses as early in life as possible, that is by using BCG.HIVA, can decrease the transmission of HIV-1 from infected mothers to infants. These BCG.HIVA-primed responses can be boosted by MVA.HIVA and/or through natural exposure to HIV-1 in breastmilk. Furthermore, the MVA.HIVA boost can be combined with MVA.85A to also increase the protection against TB[17]. Thus, this approach can serve as a vaccine platform providing infants a dual protection against both TB and HIV-1. The Sukuta Clinical Trial Protocol herein is the first phase of the overall effort towards evaluation of the BCG.HIVA-MVA.HIVA prime-boost regimen in infants born to HIV-1-infected mothers. The Sukuta Clinical Trial will test safety and immunogenicity of MVA.HIVA alone in infants born to healthy mothers, and any possible gross interference of the MVA.HIVA with the vaccines of the EPI and vice versa. The trial will not be powered to detect small interference effects.

2.2 The investigational MVA.HIVA Vaccine

2.2.1 MVA.HIVA design

The live attenuated MVA.HIVA is a recombinant MVA that contains the coding sequence of immunogen HIVA. The HIVA immunogen consists of consensus HIV-1 clade A gag p24/p17 sequences and a string of $CD8^+$ T cell epitopes including those recognized by mouse and rhesus macaque T cells, and a monoclonal antibody epitope tag [18]. The non-recombinant MVA was provided by Professor A. Mayr. The recombinant MVA was constructed by double recombination, using the β - galactosidase gene as marker. The HIVA is expressed from the late viral promoter P7.5 of vaccinia virus.

The most common local circulating strain in West Africa is a clade AG recombinant. A detailed analysis of the AG recombinant sequence shows that almost the whole of gag is accounted for by clade A. Indeed, p24 is 80% conserved across all HIV-1 clades due to structural / functional constraints, and many of the cytotoxic T lymphocyte (CTL) epitopes are shared among clades. Therefore this vaccine should be highly appropriate for the local circulating strains, and for the overlapping Gag peptides being tested in the *in vitro* assays. Indeed, positive IFN-γ ELISPOT responses were elicited using either MVA.HIVA-infected or HIVA peptide-pulsed peripheral blood mononuclear cells (PBMC) in HIV-1-infected and exposed

uninfected infants in Nairobi [19]; and the vaccine boosted HIV-1-specific responses in individuals in Oxford infected with diverse HIV-1 clades [20, 21].

2.2.2 Pre-clinical studies with MVA-vectored recombinant vaccines

The immunogenicity of MVA-based recombinant vaccines in animals is well documented. Recombinant MVA vector vaccines alone or in combination with other vectors elicit insert-specific CTL responses in mice [22-24] and rhesus monkeys [25-27], decrease plasma viraemia and increase survival [27, 28] after challenge with pathogenic SIV. Several studies in macaques have demonstrated that, although CTL responses were detected following immunization with MVA-SIV recombinants, no animals were completely protected from infection upon challenge with pathogenic SIV. However, vaccinated animals had lower virus loads and prolonged survivals compared with control animals that received only non-recombinant MVA [27-29].

The safety of recombinant MVA was studied in immune-suppressed macaques. Eight macaques were vaccinated with MVA by three different routes (intradermally, intramuscularly and intranasally) after immune-suppression by total body irradiation, anti-thymocyte globulin treatment or measles virus infection. No clinical, haematological or pathological abnormalities related to MVA inoculation were observed during a 13-day follow-up period [30].

2.2.3 Pre-clinical safety and immunogenicity of MVA.HIVA

The MVA.HIVA vaccine has been developed as a component of a DNA prime-recombinant MVA boost regimen. Pre-clinical safety studies with the pTHr.HIVA DNA and MVA.HIVA vaccines were carried out in compliance with Good Laboratory Practice (GLP) at Huntingdon Life Science, UK. A combined protocol of persistence, distribution and toxicity of the pTHr.HIVA and MVA.HIVA vaccines in the BALB/c mouse was performed, which demonstrated that the vaccines were non-toxic and detectable beyond 5 weeks after administration only in the sites of injection [31]. These results formed a basis for the approval of phase I safety and immunogenicity clinical trials in healthy HIV-1-uninfected volunteers. To support studies in HIV-1-infected subjects, toxicity and biodistribution of MVA.HIVA in mice with severe combined immunodeficiency (SCID) and SIV-infected rhesus macaques were carried out, which demonstrated that the MVA.HIVA vaccine was non-toxic in mice and non-persistent in places other than the injection site in mice and monkeys [32].

T cell immunogenicity of both the pTHr.HIVA and MVA.HIVA vaccines alone and in prime-boost combinations have been demonstrated extensively in mice [18, 31, 33-38] and rhesus macaques [26, 32, 37, 39].

2.2.4 Safety of recombinant MVA vaccines in humans

Poxviruses have played an important role in the field of vaccinology ever since Edward Jenner in 1798 protected humans against smallpox (variola) by inoculation of the related cowpox virus. MVA, a highly attenuated strain of vaccinia virus and a member of the *Orthopoxvirus* genus in the family of *Poxviridae*, made its debut towards the end of the smallpox eradication campaign as a safer alternative vaccine and has a good safety record [40]. It has been administered to more than 120,000 vaccinees as part of the smallpox eradication programme, with no reported adverse effects, despite the deliberate vaccination of high risk groups [41]. There are now more safety data from a number of recombinant MVA based vaccines expressing

antigens for hepatitis B, malaria, HIV, and melanoma from Phase I/II trials both in the UK and Africa [42-44].

Preliminary data from MVA85A vaccine [17] tested in infants in Sukuta for its immunogenicity and interference with other EPI vaccine suggest that it is safe and immunogenic (Martin Ota, personal communication). However, a preliminary analysis shows that co-administration of MVA85A with the third dose of DTwPHib / HepB caused a borderline decrease in the number of children with protective levels of Hib Abs (>0.5) compared to those who received EPI alone alone (p = 0.06). This effect was no longer apparent when a cutoff for protective Abs of >1.0 was used, in line with the UK National Vaccine Evaluation Consortium value for protective levels (p = 0.39). Antibody responses to diphtheria, tetanus and hepatitis B were not adversely affected by MVA85A. As a result of these data it has been decided not to administer MVA.HIVA with EPI vaccines.

2.2.5 Safety of MVA.HIVA in adult humans.

The complete list of clinical studies using the MVA.HIVA vaccine is given in Table 1. All these trials were performed prior to introduction of the EudraCT numbering system. In summary, the MVA.HIVA vaccine was used in 13 phase I and II preventive clinical trials and some therapeutic studies [45], in which 375 subjects received 741 doses of MVA.HIVA at four dosage levels ranging from 5×10^6 to 2.5×10^8 plaque-forming units using three routes of administration (i.d. i.m, s.c.). MVA.HIVA was given alone or as boost to pTHr.HIVA DNA. No SAE's definitely or probably related to rMVA.HIVA have been reported. In healthy subjects, the short- and medium-term safety profiles in these studies indicate that the MVA.HIVA vaccine is generally safe and well tolerated. Following intradermal vaccination local redness and discomfort were frequently reported, with a few vaccinees developing a crust and scab. Reactogenicity peaked at 3 days post-vaccination, resolved spontaneously by 2 weeks and was milder for a second dose of the vaccine. One volunteer in the IAVI 003 trial experienced an SAE 2 days after the first vaccination, which involved fever of 38.5 ^oC, malaise, myalgia, nausea and headache. This volunteer was hospitalised for 2 days, for observation and rehydration. The fever and vomiting resolved within 48 hours and the malaise 2 days later. The SAE fully resolved 10 days after onset. The event was judged as possibly related to the vaccine and this volunteer did not receive the second dose of MVA.HIVA. The same volunteer had similar complaints again 2 months later despite discontinued vaccination. During this second event, several individuals had similar complaints at the hospital where the volunteer worked as a nurse and investigations suggested an epidemic of a viral infection caused by the small round-structure virus (SRSV). The relationship between the adverse event and the vaccine remains unclear [46]. Vaccination of HIV-1-infected patients on HAART with MVA.HIVA was well tolerated and no serious adverse events were observed. $CD4^+$ T cell counts remained stable and viral loads were undetectable throughout the follow up, which was for one year after the first immunization [20, 21]. It should be noted that in most of these studies MVA.HIVA was administered intradermally, but in the Clinical Protocol PV001 it will be delivered intramuscularly. This is expected to decrease the local skin reactivity.

Table 1. Completed trials involving MVA.HIVA vaccination.

Trial No.	Location	No.	HIV	Total No.

		Subjects Receiving MVA.HIVA	Status	of Doses in Trial
IAVI 003	Oxford	8	-	15
IAVI 005	Oxford	9	-	18
IAVI 004	Nairobi	12	-	21
IAVI 002/8	Nairobi	10	-	20
IAVI 006	Oxford/St. Mary's, London	108	-	216
IAVI 009	Uganda	40	-	80
IAVI 010	Nairobi/St. Thomas, London	90	-	180
IAVI 011	Lausanne/PHRU-Soweto/MRC Durban/ Simbec-Wales	66	-	132
IAVI 016	Oxford	16	-	24
Ther 002	Oxford	8	+	16
Ther 003/4	Oxford	8	+	19
**Total**		**375**		**741**

2.2.6 Immunogenicity of MVA.HIVA in clinical trials

MVA.HIVA vaccine delivered alone and in a prime-boost regimen was tested in several hundred healthy and HIV-1-infected volunteers in Europe and Africa, and the clinical immunogenicity results are summarized in a recent publication [45]. Overall these trials demonstrated that MVA.HIVA vaccine is less efficient in priming HIV-1-specific responses, but it can deliver a consistent boost to both $CD4^+$ and $CD8^+$ T cells, which is particularly strong if the T cell responses are well primed, e.g. HIV-1 infected patients [20, 21, 47]. Thus, in the last trial in healthy volunteers, MVA.HIVA alone primed responses were not detectable using the ex vivo IFN-γ ELISPOT assay, but in cultured IFN-γ ELISPOT assays, HIV-1-specific memory T cells were found in 5 out of 8 volunteers. When primed with a pTHr.HIVA DNA vaccine, MVA.HIVA induced detectable memory HIV-1-specific predominantly CD4 T cell responses in 8 out of 8 volunteers [48]. In HIV-1 infected patients on highly active antiretroviral treatment, a significant amplification and broadening of $CD8^+$ and $CD4^+$ IFN-γ responses to vaccine-derived epitopes was observed in all (n = 16) vaccinees, but not in unvaccinated controls (n = 2), in the absence of rebound viraemia. After MVA.HIVA administration, vaccine-expanded $CD8^+$ T cells identified by tetramer reactivity were transiently activated and had upregulated perforin levels. Expansions persisted for at least one year and consisted predominantly of either $CD45RA^-$ $CCR7^+$ or $CD45RA^-$ $CCR7^-$ $CD8^+$ T cells. Increased frequencies of $CD4^+$ T cells expressing intracellular IL-2 and IFN-γ were noted in several vaccinees [21].

2.2.7 Containment and transmission of live MVA-vectored vaccines

The live MVA vector does not replicate in human cells and therefore is not transmissible between individuals. To prevent leakage of recombinant MVA following intramuscular injection, the injection site will be covered with a dry adhesive dressing 10 minutes after the vaccination for at least 50 minutes.

The UK Health and Safety Executive (HSE) has previously classified MVA.HIVA as a Class I Genetically Modified Organism (GMO). An SOP regarding

the contained use of Genetically Modified Organisms has been developed following recommendations of the Gene Therapy Advisory Committee (GTAC), UK.

2.3 Potential Risks and Benefits

2.3.1 Potential Risks

The general risks to participants in this Phase 1 study are associated with phlebotomy and with vaccination. The total volume of blood drawn over the study period (20 mL) should not compromise these otherwise healthy infants. It is important to note that MVA has been administered to over 120,000 subjects as part of the smallpox campaign with no serious adverse reactions, and therefore has a good safety record. Potential risks include the following:

Local reactions: Mild tenderness and bruising may result from venepuncture. An inflammatory reaction as manifested by redness, swelling, scaling and/or tenderness may occur at the site of vaccine injection. In previous studies with MVA.HIVA, these local reactions resolved spontaneously within a couple of days.

Systemic signs and symptoms: Systemic events following administration of MVA as a vector that could potentially occur include a flu-like illness with low-grade fever, chills and malaise. As with any other vaccine, temporary ascending paralysis, the Guillain-Barré syndrome (GBS) or immune mediated reactions that can lead to organ damage may occur. For influenza vaccines an excess of approximately 1 GBS case per million persons immunized has been observed. No cases were observed in people under 45 years of age. However, GBS has never been known to be caused by any recombinant MVA or vaccines containing any of their components.

Allergic Reactions and Anaphylaxis: As with any vaccine, allergic reactions are possible.

All subjects will be closely monitored and followed up.

2.3.2 Known Potential Benefits

This is a preliminary study with the overall goal of proving that a vaccine can protect against mother-to-child transmission of HIV-1 infection in humans. There are no proven data to suggest that infants will benefit directly from participation in this study by being protected against HIV-1. However, it is hoped that the information gained from this study will contribute to the development of a safe and effective HIV-1 vaccine for prevention of MTCT of HIV-1 through breast milk, and thus long term foreseeable benefits for these children when they reach child bearing age and for their children can be envisaged.

All participants will receive their EPI vaccines and free medical health care at Sukuta Health Centre for the first three years of life. They will also be followed up regularly at home by one of the study field workers. They will benefit by having information about their general health status, and the rigorous follow up visits could also enhance early detection and management of medical conditions that might arise in the course of

the study. Any infant with antibody titres to EPI vaccines below the level of protection will receive a booster dose of that particular vaccine.

3 OBJECTIVES

Primary:

- Safety and immunogenicity of MVA.HIVA vaccine in 20 week old healthy Gambian infants born to HIV-1/2-uninfected mothers.

Secondary:

- Gross impact of MVA.HIVA on the immunogenicity of EPI vaccines (DTwPHib, HepB, PCV-7 and OPV) when administered 4 weeks after the last EPI in infants who received BCG vaccine within the first two weeks of life. The trial will not be powered to detect small interference effects.

Endpoints:

For safety and reactogenicity: Actively and passively collected data on adverse events (AEs).

For immunogenicity to EPI vaccines: Antibody levels to specific vaccines.

For immunogenicity to MVA.HIVA: Frequency of IFN-γ producing cells determined in ex-vivo (effector) and 10-day cultured (memory) ELISPOT assays after overnight stimulation with pools of HIVA-derived peptides. We shall consider the MVA.HIVA vaccine to be immunogenic in infants if at least 50% of vaccinees have detectable HIV-1-specific T cell responses at any time point after the vaccination. In the cultured IFN-γ ELISPOT assay, a positive response is defined to be at least 250 SFU/10^6 cells above the no-peptide background, and at least 4x the no-peptide background. We shall be using strandardized and trial-tested assays, which have been described in Goonetilleke et al. [48]. MVA.HIVA alone is not considered to be a strong priming vaccine, but it can strongly and consistently boost HIV-1-specific T cell responses if they have been previously primed [20, 21, 47, 48]. It is planned in the future to use the MVA.HIVA vaccine as a booster to a BCG.HIVA prime, which should enhance the generation of HIV-specific T cell levels after the MVA.HIVA vaccination.

4 STUDY DESIGN

This is an open randomized phase I study evaluating the safety and immunogenicity of a single dose of MVA.HIVA vaccine administered at 20 weeks of age to infants born to HIV-1/2-uninfected mothers, and its gross impact on the immunogenicity of EPI vaccines administered not less than 4 weeks before. The immunology laboratory staff will be blinded for the study product / control group allocation.

Study site: Sukuta Health Centre, The Gambia

4.1 Sample size:

A total of 48 mother-infant couples will be enrolled into the study and randomized in two groups of 24.

Group 1: MVA.HIVA administered at 20 weeks of age (n=24)
Group 2: Normal EPI vaccine schedule (control group) (n=24)

This study is a pilot study and the immunogenicity analyses will be mainly descriptive. As such, it is not powered to provide definitive evidence of vaccine-induced effects; rather it is based on smaller numbers of participants and will only indicate trends, feasibility, or size of effect and variability of measurements to inform the design of more definitive studies. The rate of serious adverse events will be used as one measure of the safety of the candidate vaccine. Table 2 shows examples of sample size calculations for safety in terms of the ability to detect SAEs, i.e. the probabilities of observing 0, 1+ (1 or more), 2+ or 3+ SAEs among group of n=24 for a range of possible true event rates. If none of the 24 volunteers receiving the vaccine experiences an SAE definitely related to the vaccine, the 95% two-sided upper confidence bound for the rate of such reactions in the population is 13.8% (Table 3).

Table 2. Probabilities of detecting events for n=24

Event rate (SAE)	Detect. Probabili (0 events/24 vol's	Detect. Probabili (1+ events/24 vol	Detect. Probabili (2+ events/24 vol	Detect. Probabili (3+ events/24 vol
0.010	0.88	0.11	0.01	<0.01
0.025	0.74	0.26	0.03	<0.01
0.035	0.65	0.35	0.06	0.01
0.050	0.54	0.46	0.12	0.02
0.100	0.28	0.72	0.34	0.11
0.150	0.14	0.86	0.56	0.26
0.200	0.07	0.93	0.73	0.44
0.250	0.03	0.97	0.84	0.61

Table 3. 95% CI for the probability of SAE

Observed SAE (n=24)	95% Confidence Interval
0/24	0.0 – 13.8%
1/24	0.7 – 20.2%
2/24	2.3 – 25.8%
3/24	4.3 – 31.0%
4/24	6.7 – 35.9%
5/24	9.2 – 40.5%

4.2 Randomisation

Infants who have been sensitised into the study will be invited to attend Sukuta Health Centre at 19 weeks of age. Those with a documented BCG vaccination within the first two weeks of life who have received all their EPI vaccines on schedule at the Sukuta Health Centre will be eligible for recruitment. Parents of those infants who meet all inclusion and none of the exclusion criteria will be asked to sign the informed consent form. A baseline blood sample will be taken and they will be asked to return to the Health Centre at 20 weeks of age. When they return for their 20 week visit the blood results will be checked and providing they still meet the inclusion and exclusion criteria they will be randomised to one of the two study groups of 24 (Table 4) by picking the next envelope (block randomised). The sensitisation number and allocation to vaccine group will be entered into the enrolment log. This will assign them to one of the two treatment groups: Group 1 will receive MVA.HIVA and Group 2 will not receive any vaccines. Infants recruited into this study will receive their routine EPI and study vaccines from the study team in order to reduce confounding variables that might result from variations in age at vaccination, and batch of vaccines administered.

Table 4. Study groups

Group	n	Treatment
1	24	$5x10^7$ pfu of MVA.HIVA i.m.
2	24	No vaccine (Control)

Masking
It will not be a blinded study, however, the personnel who are performing the immunology assays (primary outcome measures) will be masked as they will be dealing with coded laboratory specimens and not know the allocation to groups.

5 STUDY POPULATION

All subjects must meet all of the inclusion criteria and none of the exclusion criteria to participate in this study.

5.1 Inclusion Criteria

a. Healthy infants, 19 weeks of age, with weight for age z-scores within 2 standard deviations of normal.
b. Have received all standard EPI immunizations according to national immunization programme (Table 5).
c. Written informed consent by parent.
d. Mother HIV-1/2-uninfected.

Table 5. Overview of EPI vaccination schedule in The Gambia in the first 12 months of life

Age	
Birth	BCG, OPV, HepB
8 w	OPV, Pentavalent, PCV-7
12 w	OPV, Pentavalent, PCV-7
16 w	OPV, Pentavalent, PCV-7
9 m	Measles, Yellow fever, OPV

5.2 Exclusion Criteria

a. Acute disease at the time of vaccination (acute disease is defined as the presence of a moderate or severe illness with or without fever). All vaccines can be administered to persons with a minor illness such as diarrhoea, mild upper respiratory tract infection with or without low-grade febrile illness, i.e. axillary temperature of <37.5 °C).
b. Axillary temperature of ≥ 37.5 °C at the time of vaccination.
c. Any clinically significant abnormal finding on screening from biochemistry or haematology at 19 weeks.
d. History of allergic disease or reactions likely to be exacerbated by any component of the vaccine, e.g. egg products.
e. Presence of any underlying disease that compromises the diagnosis and evaluation of response to the vaccine.
f. Invasive bacterial infections (pneumonia, meningitis).
g. Any other on-going chronic illness requiring hospital specialist supervision.
h. Administration of immunoglobulins and/or any blood products within one month preceding the planned administration of the vaccine candidate.
i. Any history of anaphylaxis in reaction to vaccination.
j. Research physician's assessment of lack of willingness by parents to participate and comply with all requirements of the protocol, or identification of any factor felt to significantly increase the infant's risk of suffering an adverse outcome.
k. Likelihood of travel away from the study area.
l. Untreated malaria infection.
m. Any other clinical evidence of infection.

5.3 Sensitisation and Recruitment Procedures

Field workers will approach women shortly after delivery at Sukuta Hospital and provide them with study information sheet in a language that they understand. Women will be encouraged to discuss the study with their husbands and involve the husband in the decision making process. The study information sheet will provide full details of the study procedures and what will be required of them if they participate. The information sheet will include: the purpose of the study and what it will involve for the infants will be described, including the study screening and the informed consent procedure, methods of vaccination, observation details, spectrum of likely side effects, possible adverse reactions, follow-up details and extent of blood sampling, as well as risks of vaccination and the unproven benefits of vaccination. The need for an HIV vaccine including a simple picture of the burden of HIV on the community, country and Africa, the current status of HIV-1 vaccine development including the fact that it is likely to be a prolonged process, will be explained. It will be stressed that this is an experimental vaccine and it cannot be guaranteed to provide protection. The need for a health screening before receiving vaccinations

because the vaccine is in an early stage of development will be explained. The information sheet will have been submitted to and willl have received approval from the local ethics committee.

Parents must understand that these vaccines cannot cause HIV / AIDS.

Parents must understand that the MVA.HIVA vaccine has not been shown to prevent HIV infection and this will be stressed during the recruitment stage.

As it is an MRC policy, study parents are told that they cannot be a subject in a vaccine trial or any drug trial whilst they are a participant in this study.

Women, and their husbands if present who have been sensitised, will be invited to return for a screening visit when their child has reached 16 weeks of age. If they are still interested in participating in the study, they will be invited to ask questions, and when fully satisfied with all the answers will be asked to read and sign (or thumb-print) the informed consent document which will have been approved by the combined Gambian Government / MRC Joint Ethics Committee. Consent must be obtained prior to any study-related evaluations being performed (see consent check list, appendix). One copy of the signed consent form will be given to the parents, and a second copy will be retained by the investigator. Those infants whose parents have signed the consent document will be assessed at Sukuta health centre as described below in the study schedule.

It will be stressed that the infants can be withdrawn from the study at any stage.

6 STUDY ENROLMENT AND SCHEDULE OF FOLLOW-UP VISITS

Infants who meet all inclusion and none of the exclusion criteria will be assigned a 3 digit study identification number (sensitisation number) if the parents have expressed an interest in the child participating in the study. They will retain this number for the duration of the study including at randomization. The consent form will need to be signed before the first study intervention at the 16 week visit (Visit 1). The randomisation into one of the treatment groups will be performed at their 20 week visit (Visit 3) (Table 6).

Infants recruited into this study will receive their routine EPI and study vaccines from the study team in order to reduce confounding variables that might result from variations in age at vaccination, and batch of vaccines administered.

All Gambian infants are issued with a blue health card at birth. A sticker indicating that the child is involved in an MRC study and should only receive EPI vaccines from the study team at Sukuta Health Centre up to the age of 9 months will be placed on the blue health card. This sticker will be removed when the child ceases to be in the study (i.e. end of the study period, not eligible for randomisation or dropped out). Table 6 illustrates the sequence of events from recruitment at birth, through to MVA.HIVA vaccination and the subsequent follow-up visits (explained in more detail in the text that follows):

CONFIDENTIAL

Table 6. Sequential events from delivery through vaccination & follow-up visits

Age of study infants	16w	19w	20w	21w	28w	36w
Study visit	1	2	3	4	5	6
Bleeding schedule relative to study product administration		-1w		+1w	+8w	+16w
Group 1 (n=24)	EPI	**Bleed**	**MVA.HIVA**	**Bleed**	**Bleed**	**Bleed** EPI **ESP**
Group 2 (n=24)	EPI	**Bleed**		**Bleed**	**Bleed**	**Bleed** EPI **ESP**

Bleed – 5 mL of blood; EPI = Vaccines of Expanded Programme on Immunization; **ESP** = End of study protocol; w = week

6.1 Laboratory Tests

Haematology Full blood count (FBC) including platelet count
Biochemistry Creatinine, alanine transaminase (ALT), alkaline phosphatase (ALP), bilirubin
MVA.HIVA immunogenicity Frequency of IFN-γ producing cells using ex-vivo and cultured ELISPOT assays after overnight stimulation with pools of 15-mer peptides overlapping by 11 amino acids spanning the length of antigen HIVA.
EPI immunogenicity Measurement of vaccine-induced antibody levels to DTwP, Hib, HepB, PCV-7 and OPV.

Table 7. Temporal course of infant blood investigations

Investigation	Wk 19	Wk 21	Wk 28	Wk 36
Haematology	X	X	X	
Biochemistry	X	X	X	
MVA.HIVA Immunogenicity	X	X	X	X
EPI Immunogenicity	X	X		
HIV test			X	
HLA typing				X

Dissemination and explanation of blood results

All non-immunology blood results will be given and explained to all parents after screening and at follow-up visits. Immunology results will be explained in general terms, for the group and not for individuals, at the end of the study. Those with abnormal blood results at screening will be offered appropriate investigations and treatment or referral as necessary. Parents will receive an explanation of all screening results before proceeding with MVA.HIVA immunization.

6.2 Study Visits

CONFIDENTIAL

Sensitisation and Informed Consent: The majority of children will be sensitised at birth or in the 1st few weeks of life. If sensitised at birth the study will be explained after the mother has had sufficient time to recover from the labour, and parents will be invited to ask questions. If the parents express an interest in taking part in the study their child will be assigned a 3-digit sensitisation number and told that they will be invited to return to the clinic when their child has reached 16 weeks of age to discuss their interest and potential eligibility for participation in the study. If they are found to be willing and eligible at this visit, parents will be asked to sign/thumbprint the informed consent form. The research physician must be available to answer any questions during the informed consent process, and is the person designated to sign the informed consent form. The consent form must also be signed by an independent witness who will be present during the consent process. The mother will undergo voluntary counselling and testing (VCT) for HIV by the trained study physician at Sukuta Health Centre. A VCT trained field worker will be available to translate during this process in a language that the mother understands.

HIV Positive Women: Any women who tests positive for HIV will be followed up and managed for their HIV at Sukuta Health Centre where practicable for 3 years. Her child will be offered an HIV PCR test and treatment and follow up as appropriate. Sukuta Health Centre will act as the first point of clinical contact for those women found to be HIV infected during this study, however they will also need to enrol into a government accredited HIV clinic as part of the National HIV Programme. The choice of clinic will be decided on a case-by-case basis in consultation with the mother according to their situation and preference.

Visit 1 – SCREENING VISIT (Infant aged 16 weeks ± 10 days)

- Subjects will be brought to the vaccine trial clinic.
- Consent to participate in the study will be obtained.
- An interim medical history will be taken.
- Physical examination including vital signs (temperature, respiratory rate, pulse rate), and anthropometry (weight, mid left upper arm circumference (MUAC), length) will be performed by the research field worker / research nurse.
- EPI vaccines appropriate for age will be given (Pentavalent, PCV-7 and OPV).
- Appointment given for 3 weeks time.

Visit 2 – BASELINE VISIT (Infant aged 19 weeks ± 10 days)

- Subjects will be brought to the vaccine trial clinic.
- Continued consent to participate in the study will be confirmed.
- Inclusion and exclusion criteria will be checked.
- An interim medical history will be taken.
- Physical examination including vital signs (temperature, respiratory rate, pulse rate), and anthropometry (weight, mid left upper arm circumference (MUAC), length) will be performed by the research field worker / research nurse.
- 5 mL of venous blood will be collected for baseline FBC, biochemistry, pre-MVA.HIVA immunogenicity and EPI vaccine antibody assays.
- Appointment given for 1 week time.

Visit 3 – VACCINATION VISIT, MVA.HIVA vaccination day (Infant aged 20 weeks ± 10 days)

- Subjects will be brought to the vaccine trial clinic.
- Continued consent to participate in the study will be confirmed.
- Inclusion and exclusion criteria will be checked.
- Results of FBC and biochemistry from previous week will be reviewed by the research physician to ensure the child is eligible for randomisation.
- An interim medical history will be taken.
- Physical examination including vital signs (temperature, respiratory rate, pulse rate), and anthropometry (weight, mid left upper arm circumference (MUAC), length) will be performed by the research field worker / research nurse.
- The screening safety profile will be reviewed by the research physician and those children that fulfill all inclusion criteria and no exclusion criteria will be randomised to one of the two study groups by selecting an envelope containing the block randomized treatment group that they are in:
    - **Group 1** infants will receive MVA.HIVA (5 x 10^7 pfu in no more than 100 µl) intramuscularly into the deltoid muscle of the left arm with a BCG scar. The injection site will be covered after 10 minutes with a sterile dressing that will be removed at the one-hour observation.
    - **Group 2** (control) will not receive any vaccines.
- The infant and parent will remain at the clinical area for one hour following vaccination and will then be allowed to return home.
- The injection site will be inspected at 10 minutes when a dressing will be applied.
- Vital signs will be checked at 30 minutes (+/- 5 min) and 1 hour (+/-10 min) post injection.
- At 1 hour (+/-10 min) the dressing will be removed and discarded. The largest diameter of any induration or redness and any other local reaction at 1 hour will be documented into the CRF. An assessment for possible systemic adverse events (AE's) will be carried out and documented on the CRF, and any concomitant medication documented.
- A nurse / field worker will visit subjects at home daily for the first two days after MVA.HIVA vaccination to record safety and reactogenicity data on a standard CRF form.

Contraindications to Vaccination

See section 5.2

Visit 4 – FOLLOW-UP VISIT Week 1 (±2 days) post vaccination (Infant aged 21 weeks ± 10 days)

- Subjects will be brought to the vaccine trial clinic.
- Continued consent to participate in the study will be confirmed.
- An interim medical history will be taken.
- Physical examination including vital signs (temperature, respiratory rate, pulse rate), and anthropometry (weight, mid left upper arm circumference (MUAC), length) will be performed by the research field worker / research nurse.
- The injection site will be inspected by the research physician and local reactogenicity including the largest diameter of any induration or redness will be documented into the CRF.
- Adverse events that may have occurred since the infant was vaccinated and any concomitant medications will be documented in the CRF.

- 5 mL of venous blood will be collected for FBC, biochemistry, MVA.HIVA immunogenicity and EPI vaccine antibody assays.
- Appointment at the clinic will be given for 7 weeks time.

Visits 5 – FOLLOW-UP VISIT, Week 8 (± 1 week) post vaccination (Infant aged 28 weeks ± 10 days)

- Subjects will be brought to the vaccine trial clinic.
- Continued consent to participate in the study will be confirmed.
- An interim medical history will be taken.
- Physical examination including vital signs (temperature, respiratory rate, pulse rate), and anthropometry (weight, mid left upper arm circumference (MUAC), length) will be performed by the research field worker / research nurse.
- The injection site will be inspected by the research physician and local reactogenicity including the largest diameter of any induration or redness present will be documented into the CRF.
- Adverse events (local and systemic) that may have occurred since the infant was vaccinated and any concomitant medications will be documented in the CRF.
- 5 mL of blood will be collected for HIV testing, FBC, biochemistry and immunogenicity of MVA.HIVA.
- Any child that has a developed a positive HIV antibody test as a result of the vaccine will be recalled and tested for HIV by PCR. Assuming they have a negative PCR test, they will have a repeat HIV antibody test at the 9 month visit. If it is still positive at 9 months the test will be repeated every 6 months until they test negative for HIV antibodies.

Visit 6 – FOLLOW-UP VISIT/END OF STUDY, age of 9 months (Infant aged 36 ± 2 weeks)

- Subjects will be brought to the vaccine trial clinic.
- Continued consent to participate in the study will be confirmed.
- An interim medical history will be taken.
- Physical examination including vital signs (temperature, respiratory rate, pulse rate), and anthropometry (weight, mid left upper arm circumference (MUAC), length) will be performed by the research field worker / research nurse.
- The injection site will be inspected by the research physician and findings documented in the CRF.
- Adverse events (local and systemic) that may have occurred since the last visit and any concomitant medications will be documented in the CRF.
- 5 mL of blood will be collected for immunogenicity of MVA.HIVA and HLA typing.
- EPI vaccines appropriate for age and study group will be given (measles vaccine, yellow fever, OPV)
- Any child that was HIV antibody positive at 28 weeks of age will be re-tested. If the test remains positive they will be given an appointment for repeat testing every 6 months until they become HIV antibody negative.
- Parents will be told that their babies have graduated from the study. Study sticker will be removed from their health cards and children will continue to be offered free health care at Sukuta Health Centre until 3 years of age.

7 STUDY INTERVENTION / INVESTIGATIONAL PRODUCT

Study Product Acquisition
Study product will be shipped by IDT Biologika GmbH, Am Pharmapark, D-06861 Dessau-Rosslau, Germany directly to the trial site.

Formulation, Packaging and Labelling
MVA.HIVA is manufactured to Good Manufacturing Practice conditions by Impfstoffwerk Dessau-Tornau (IDT), Germany, as contracted by UK's Medical Research Council. It is supplied as a ready to use solution in glass vials. These vials are labelled as containing MVA.HIVA in accordance with good manufacturing practice guidelines on investigational medicinal products.

Product Storage and Stability
MVA.HIVA will be stored in an allocated freezer at or below –20° C in the locked freezer room at MRC Fajara. On the day of administration it will be placed in a cold box containing dry ice and transported to the study site where it will be stored in the cold box until thawing for use. The study physician will be in custody of the box at all times.

Preparation, Administration and Dosage of Study Intervention/Investigational Product
The batch of the vaccine, MVA.HIVA, will be provided in vials of 200 µl at a concentration of no less than 5x10^{8} pfu/ml in 10 mM Tris buffer, 0.9% NaCl. The dose of MVA.HIVA to be used in this stage will be 5x10^{7} pfu.

On the vaccination day, vaccines will be transported to Sukuta Health Centre in a cold box containing dry ice. It will be allowed to thaw to room temperature before use, and administered within 60 minutes of thawing. One vaccine vial per subject to be vaccinated will be used. The vaccine will be administered intramuscularly over the deltoid region of the left arm (the usual site of BCG vaccination). The physician will draw up the vaccine and the volume drawn will be checked and signed for onto the CRF by the physician and one other member of staff at Sukuta Health Centre (physician / nurse or field worker). The physician will wear gloves and eye protection when drawing up and administering the vaccine. Subjects will stay in the health centre for observation for at least 60 minutes after vaccination. During administration of the vaccines, medicines and resuscitation equipment will be immediately available for the management of anaphylaxis.

In order to minimise dissemination of the recombinant vectored vaccine virus into the environment the inoculation site will be covered with a dressing 10 minutes after immunization. This should absorb any virus that may leak out through the needle track. The dressing will be removed from the injection site at the end of the 60 minute observation period and will be disposed as GMO waste by autoclaving and incineration, in accordance with the relevant SOP and current standard practice at MRC, The Gambia.

8 ASSESSMENT OF SCIENTIFIC OBJECTIVES

Specification of the Appropriate Outcome Measures

Primary Outcome Measures

- The safety of MVA.HIVA will be determined by analysis of adverse events, reactogenicity, biochemical and haematological data that will be actively collected.

- Assessment of the immunogenicity of a single dose of MVA.HIVA (administered 4 weeks after the last EPI vaccines to healthy infants who have been vaccinated with BCG) by measuring the frequency of IFN-γ-producing cells following stimulation with pooled 15-mer peptides derived from the HIVA immunogen in *ex-vivo* (effector) and 10-day cultured (memory) ELISPOT assays.

Secondary

- Determination of gross interference of MVA.HIVA with the immunogenicity of DTwPHib, HepB, OPV and PCV-7 vaccines will be through comparing actively collected data on antibody levels to the antigens contained in these EPI vaccines in the vaccine and control groups. Historical data from other studies in the same population may also be used to provide normal ranges.

- If there are any residual PBMCs after the IFN-γ ELISPOT assays have been performed, other immunological assays may be performed, which might include:

    - ELISPOT assays for interleukin 2 and tumour necrosis factor alpha
    - T cell proliferation to HIVA peptides
    - T cell depletion assays
    - Cytokine detection using ELISA/LUMINEX
    - Intracellular cytokine staining and multicolour flow cytometric analysis

9 ASSESSMENT OF SAFETY

All adverse events (AE) occurring in participants after administration of vaccine will be documented and reported as described below.

9.1 Definitions

Adverse Event

An AE is any untoward medical occurrence, including a dosing error, which may occur during or after vaccination. The AE may or may not have a causal relationship with vaccination as indicated by physical signs, symptoms, and/or clinically significant laboratory abnormalities that occur. The definition includes intercurrent illnesses, injuries, exacerbation of pre-existing conditions, and events occurring as a result of product misuse or overdose.

A change in a laboratory variable is considered an AE if it leads to a change in the subject's functional status or if it is considered by the attending physician to be

clinically significant. The following laboratory parameters will be monitored during the course of this study (as previously outlined): full blood count (FBC), creatinine and ALT.

Expected local reactions to the vaccine will not be recorded as an adverse event, but will be recorded on the CRFs. These include:

- Redness
- Induration
- Scaling / Blistering / Ulceration
- Axillary lymph node enlargement
- Pain (scored as follows)
  - 0 No pain
  - 1 Painful to touch
  - 2 Partial restriction of activities
  - 3 Unable to use the arm

Serious Adverse Event (SAE)

Any AE (whether or not considered related to the investigational product) that results in any of the following outcomes is defined as an SAE.

1) Death (i.e. death from any cause at any time)

2) Life-threatening event (i.e. the subject was, in the view of the investigator, at immediate risk of death from the event that occurred). This does not include an AE that, if it occurred in a more serious form, might have caused death.

3) Persistent or significant disability or incapacity (i.e., substantial disruption of one's ability to carry out normal life functions).

4) Hospitalisation: regardless of length of stay, even if the hospitalization is a precautionary measure for continued observation. Hospitalisation (including inpatient or outpatient hospitalization for an elective procedure) for a pre-existing condition that has not worsened unexpectedly does not constitute a serious AE.

5) An important medical event (that may not cause death, be life threatening, or require hospitalization) that may, based upon appropriate medical judgment, jeopardize the subject and/or require medical or surgical intervention to prevent one of the outcomes listed above. Examples of such medical events include allergic reaction requiring intensive treatment in an emergency room or clinic, blood dyscrasias, or convulsions that do not result in inpatient hospitalization.

9.2 Adverse Events Assessment

For every adverse event (AE), an assessment of the relationship of the event to the administration of the vaccine will be undertaken. A related AE refers to an AE for which there is a possible, probable or definite relationship to administration of a vaccine. The investigator will make interpretation of the causal relationship of the intervention to the AE in question. This interpretation will be based on the type of event, the relationship of the event to the time of vaccine administration,

and the known biology of the vaccine. The following are guidelines for assessing the relationship of administration of the study vaccine to the AE:

1. NO RELATIONSHIP:
   - No temporal relationship to study product; *and*
   - Alternate aetiology (clinical state, environmental or other interventions); *and*
   - Does not follow known pattern of response to study product

2. POSSIBLE:
   - Reasonable temporal relationship to study product; *or*
   - Event not readily produced by clinical state, environmental or other interventions; *or*
   - Similar pattern of response to that seen with other vaccines

3. PROBABLE:
   - Reasonable temporal relationship to study product; *and*
   - Event not readily produced by clinical state, environment, or other interventions *or*
   - Known pattern of response seen with other vaccines

4. DEFINITE:
   - Reasonable temporal relationship to study product; *and*
   - Event not readily produced by clinical state, environment, or other interventions; *and*
   - Known pattern of response seen with other vaccines

Every AE observed or reported from the day of MVA.HIVA injection through to the end of the trial will be recorded on the appropriate CRF.

All AEs will be followed until resolution of the signs or symptoms or laboratory changes occurs, or until a non-study related causality is assigned.

Serious Adverse Event (SAE) Reporting

In order to comply with current regulations on serious adverse event reporting the investigator pledges to document accurately the event, to respect notification deadlines, to provide the Sponsor with all necessary information and, if requested by the Sponsor, to give access to source documents. All SAEs will be reported by telephone, or email or fax to the Sponsor and Local Safety Monitor within one working day (24 hours) of the investigator becoming aware of the SAE occurrence.

Unexpected SAEs that are, in the opinion of the Principal Investigator or designated research physician, related to the administration of any of the research procedures should be reported to the local and OXTREC Ethics Committees and the Gambia Medicines Board within 15 calendar days of the PI becoming aware of the event. If the event is fatal or life threatening, the event should be reported within 7 calendar days.

Minimum details to be given in a telephone report are:

- Name of reporting doctor and contact telephone number.
- Study number.
- Nature of adverse event.
- Subject details (number, initials, sex, date of birth, weight and age).
- Date and time of event.
- Date and time of MVA.HIVA administration and dose.
- Other drug history.
- Other relevant history.
- Outcome.
- Causality.

The event will be documented on the SAE page of the CRF and reported to the Sponsor, Local Safety Monitor, DMEC, relevant Ethics Committees, Gambia Medicines Board, TSC, collaborators and funding agency as appropriate.

After the local ethics committee's response to the SAE report is received, the Sponsor, Principal Investigator, Local Safety Monitor and available co-investigators will communicate to determine the future plan for the study, which could involve amending the protocol, discontinuing the vaccinations, or continuing unchanged for the other volunteers.

A suspected unexpected serious adverse reaction (SUSAR) is different from an SAE in that it is unexpected and thought to be related to the study vaccine. If an unexpected adverse reaction to the trial vaccine is suspected, the Sponsor will comply with the regulatory requirements on reporting SUSARs.

9.3 Adverse Event Monitoring

Local Safety Monitor and Data Monitoring & Ethics Committee

The Local Safety Monitor (LSM), Dr Uduak Okomo, will provide real-time safety oversight. The LSM will review SAEs immediately after they occur and follow the events until resolution. The LSM has the power to suspend enrolment into the study if deemed necessary following an SAE and convene a meeting with the Sponsor and Principal Investigator immediately. The LSM can convene a meeting with the Sponsor, PI or other parties involved at any time for further discussion as necessary.

An independent Data Monitoring & Ethics Committee (DMEC) will be set up prior to initiating the trial. The DMEC will be appointed to review relevant safety information and all Serious Adverse Events. Immunogenicity data will also be provided to the DMEC. A detailed description of DMEC procedures is provided in the DMEC charter.

Methods and Timing for Assessing, Recording, and Analyzing Safety Parameters

The following scale will be used to assess the severity of all AEs:

0 = Absence of the indicated symptom
1 = Mild (awareness of a symptom but the symptom is easily tolerated)
2 = Moderate (discomfort enough to cause interference with usual activity)

3 = Severe (incapacitating; unable to perform usual activities; requires absenteeism or bed rest)

4 = Life-threatening

Procedures to be Followed in the Event of Abnormal Laboratory Test Values or Abnormal Clinical Findings

Any infant found to have a clinically significant condition prior to inclusion in the study (e.g. heart failure, respiratory distress) or abnormal biochemical or haematological result may have the test repeated to ensure this was not a laboratory error or spurious result. If the test remains clinically significant the infant will be excluded from the study and referred to the appropriate facility (e.g. Royal Victoria Teaching Hospital paediatric clinic) for further investigation and management with the permission of their parents. If occurring during the trial, tests may be repeated to verify the results, and further tests may be instigated to establish causality. If necessary the child will be withdrawn from the study. The infant's parents will be kept informed of all such occurrences and advised of actions to be taken, and their verbal/written consent taken for extra tests where appropriate.

Halting Rules

Any SUSAR will result in suspension of the trial until a safety review is convened. The trial may be discontinued by the Sponsor, the Gambia Government / MRC Joint Ethics Committee or the Oxford Tropical Research Ethical Committee.

The Sponsor delegates to the DMEC the right to temporarily suspend or prematurely discontinue this trial at any time and for any reason as described in the DMEC charter. If the trial is stopped or suspended prematurely, the Sponsor will inform the Principal Investigator. If such action is taken, all effort must be made to ensure the safety of the participants enrolled in the study. The Principal Investigator will inform the relevant ECs about the decision and provide the reason for the suspension or termination.

10 CLINICAL MONITORING STRUCTURE

Site Monitoring Plan

Independent trial monitors will be contracted by the UK's Medical Research Council and they will be responsible for the monitoring of this trial and compliance to ICH-GCP Guidelines. The Local Trial Monitor (LTM) of the MRC Unit in The Gambia, Ms Vivat Thomas, will be a valuable resource on-site and will work collaboratively with the Sponsor's monitor.

Pre-study Visit

A pre-study visit will be performed before the inclusion of the first infant in the study. This meeting will verify and document that the material to be used during the trial has been received and that the investigational team has been properly informed about the trial, regulatory requirements, and they are familiar with all the SOP requirements. The pre-study visit will be coordinated between the local and Sponsor monitors.

During this visit the Sponsor's monitor will run through the study flow with the

trial team alongside all the documents and ensure everyone is confident in all aspect of the trial, how it will run and how the forms need to be completed. The monitors will conduct GCP refresher training if required and also explain the safety reporting system.

Follow-up Visits

The Sponsor's monitor will carry out regular follow-up visits. The Principal Investigator will be available for these visits and should allow the monitoring staff direct access to subjects' medical files and CRFs. All trial monitors are committed to professional secrecy.

During the visits, the Sponsor's monitor will:

- carry out a quality control of trial progress: respect of protocol and operating guidelines, data collection, signature of consent forms, completion of document and appearance of SAE
- sample and product management, cold chain monitoring
- review the CRFs and correspondent correction sheets
- evaluate the number of complete or ongoing study participation events

The trial monitor shall discuss any problem with the Principal Investigator and define with him / her the actions to be taken. Once the CRFs corresponding to the last visit have been completed and signed, the investigator must be available to deal with any issues raised by the trial monitor until the database is locked.

In addition to the Sponsor's trial monitor the MRC's local monitor, Ms Thomas, will carry out regular meetings with the trial staff, where the documents and compliance to GCP will be checked, and feedback including advice and training recommendations will be given to the Principal Investigator.

The Sponsor's monitor will coordinate his / her visits with the local monitor and they will share their reports.

Close-out Visit

A close-out visit will be performed at the end of the trial by the Sponsor's trial monitors. Its goals are to make sure that:

- The centre has all the documents necessary for archiving
- All paperwork is satisfactorily completed
- The vaccine accountability is correct and all used vials have been destroyed by MRC, Gambia

Audits and Inspections

If necessary, a quality assurance audit will be carried out by independent auditors to make sure that the trial has been conducted according to the protocol and the

applicable regulations.
An inspection may be conducted by Regulatory Authorities.
The investigator must allow direct access to trial documents.

11 STATISTICAL CONSIDERATIONS

11.1 Determination of the Sample Size

No formal sample size calculation is done for the study and analyses will be mainly descriptive.

Statistical Methods
The analyses will be comparative, as clinical and laboratory data from the age-matched vaccine and untreated control groups will be compared.

11.2 Expected analyses and outcomes

11.2.1 Safety of MVA.HIVA

Determined by comparing adverse events, reactogenicity, haematological and biochemical data from the MVA.HIVA recipients and controls. See Tables 2 and 3 for the probabilities of detecting events given the current trial design.

11.2.2 Immunogenicity of MVA.HIVA

Determined by comparing between Groups 1 and 2 the frequency of IFN-γ-producing cells (by *ex vivo* and cultured ELISPot assay) in response to 15-mer peptides overlapping by 11 amino acids spanning the length of HIVA. The MVA.HIVA vaccine will be considered immunogenic in infants if at least 50% of vaccinees have detectable HIV-1-specific T cell responses at any time point after the vaccination.

11.2.3 Immunogenicity of EPI vaccines

The MVA.HIVA will be given at week 20 (4 weeks after the last EPI vaccines) so as to avoid the possibility of vaccine interference. However, vaccine antibody levels will be measured one week before and one week after MVA.HIVA (i.e. 5 weeks after the last EPI vaccines) to assess for gross interference of the MVA.HIVA vaccine with the immunogenicity of the EPI vaccines. Antibody responses to the EPI vaccines will be compared between Groups 1 and 2, with particular focus on the immunogenicity of Hib, which is the least immunogenic of the EPI vaccines and for which antibody levels below 0.5 micrograms per litre are considered unprotective.

11.3 Data Management

Mehedi Shams-Rony will be the data manager with overall responsibility for receiving, entering, cleaning, querying, analysing and storing all data that accrues from the study. The data will be entered into the subjects' CRFs and will be transferred by double entry with verification to a secure database.

12 SOURCE DOCUMENTS AND ACCESS TO SOURCE DATA/DOCUMENTS

The investigators will maintain appropriate medical and research records for this trial, in compliance with ICH GCP Guidelines and regulatory and institutional requirements for the protection of confidentiality of subjects. The Principal Investigator, co-investigators and clinical research nurses will have access to records.

The investigators will permit authorized representatives of the Sponsor, Ethics Committees, independent auditors and regulatory agencies to examine clinical records for the purposes of quality assurance reviews, audits and evaluation of the study safety and progress.

All protocol required information will be collected in Case Report Forms (CRF) designed by the Principal Investigator in collaboration with the data manager. All source documents e.g. blood result forms, radiology reports, copies of EPI vaccination cards will be filed separately and be available for review if needed.

All data on the CRFs must be legibly recorded in blue or black ink or typed. A correction should be made by striking through the incorrect entry with a single line and entering the correct information adjacent to it. The correction must be initialed and dated by the investigator or a designated, qualified individual.

Any requested information that is not obtained as specified in the protocol should have an explanation noted on the CRF as to why the required information was not obtained.

13 QUALITY CONTROL AND QUALITY ASSURANCE

Data will be evaluated for compliance with protocol and accuracy in relation to source documents. The study will be conducted in accordance with procedures identified in the protocol. SOPs will be used at all clinical and laboratory sites. Regular monitoring will take place according to ICH-GCP Guidelines, and an independent audit may be performed. Following written standard operating procedures, the monitors will verify that the clinical trial is conducted and data are generated, documented (recorded), and reported in compliance with the protocol, GCP, and the applicable regulatory requirements. The clinical trial site will provide direct access to all trial related facilities, source data/documents, and reports for the purpose of monitoring and auditing by the Sponsor, and inspection by local and regulatory authorities.

14 ETHICS/PROTECTION OF HUMAN SUBJECTS

This protocol will be approved by the Gambia Government / MRC Joint Ethics Committee, Oxford Tropical Research Ethics Committee, the Sponsor (MRC-UK), Stockholm Regional Ethics Committee, International Aids Vaccine initiative and the funder (EDCTP) before this study commences. This trial will be conducted in accordance with the current revision of the Declaration of Helsinki, and with the International Conference on Harmonisation Good Clinical Practice (ICH-GCP)

guidelines (E6) and regulations.

14.1 Informed Consent Process

Written informed consent will be obtained from a parent of all infants in the study. Fathers will be actively involved in the consent process. The consent form to be used will have been approved by the relevant ethics committees. The information sheet and consent form will be translated into Mandinka and Wolof to aid the consent process, and the translated versions will be back translated by an independent translator to ensure accuracy of the translation. Translated information sheets will be made available to the study participants as required. For those parents without fluent English language skills, a field worker who speaks their language will be assigned for all visits and an assigned impartial witness will observe the consent process in accordance with ethical requirements. The subjects may withdraw consent at any time throughout the course of the trial. A copy of the informed consent document will be given to the parents for their records. The rights and welfare of the infants will be protected by emphasizing to the parents that the quality of their medical care will not be adversely affected if they decline to participate in this study.

14.2 Subject Confidentiality

All records will be kept in locked filing cabinets, and accessed only by the investigators and the study nurses / field workers. The CRFs will record the infant's 3-digit identification number and initials, and the subject's name will not appear on the form. All computer entry and networking programs will be done with coded numbers and initials only. Only the investigator's team, the trial monitors and representatives of the Sponsor, Ethics Committees, independent auditors and regulatory authorities will have access to the records. Photographs taken of vaccination sites (with the subject's written informed consent) will not include the infant's face and will be identified by the infant's 3-digit identification number only. Digital photographs will be stored as confidential records as above. This material may be shown to other professional staff or be used for educational purposes, or be included in a scientific publication. Every effort will be taken to maintain confidentiality.

14.3 Biohazard Containment

As the transmission of blood-borne pathogens can occur through contact with contaminated needles, blood, and blood products, appropriate blood and secretion precautions will be employed by all personnel involved in the drawing of blood and shipping and handling of all specimens for this study, according to the MRC safety manual. Note that only infants born to HIV-1/2-uninfetced mothers will be enrolled.

15 DATA HANDLING AND RECORD KEEPING

The database manager will be responsible for receiving, entering, cleaning, querying, analysing and storing all data that accrues from the study. He will be responsible for linking the epidemiological and clinical data from the field and the clinic with the laboratory data from the immunology, microbiology, biochemistry, haematology and genetics laboratories. Laboratory data will be transferred directly to the database from the source document (or electronically if available), and immunological laboratory data. may be entered directly from an electronic source e.g ELISpot

results from the ELISpot reader. This process will be overseen by the Pricipal Investigator.

The study protocol, documentation, data and all other information generated will be held in strict confidence. No information concerning the study or the data will be released to any unauthorized third party, without prior written approval of the Sponsor.

Study Records Retention

Essential documents will be retained for at least 20 years according to current MRC research guidelines. These documents will be retained for a longer period however if required by the applicable regulatory requirements or by an agreement with the Sponsor. It is the responsibility of the Sponsor to inform the investigator/institution as to when these documents no longer need to be retained

Protocol Deviations

The Principal Investigator (PI) will conduct the trial in compliance with the protocol agreed to by the Sponsor and which was given approval by the Ethics Committees. The Principal Investigator will sign the protocol to confirm agreement.

The PI will not implement any deviation from, or changes of the protocol without agreement by the Sponsor and prior review and documented approval from the relevant Ethics Committees of an amendment, except where necessary to eliminate an immediate hazard(s) to trial subjects, or when the change(s) involve only logistical or administrative aspects of the trial (e.g. change in monitor(s), change of telephone number(s)).

The PI or a person designated by the PI will document any deviation from the approved protocol on the CRF, where appropriate, and record and explain any deviation in a file note that will be maintained as an essential document. Deviations from the protocol, GCP or trial specific requirements that might have an impact on the conduct of the trial or the safety of participants will be reported within 5 working days to the Sponsor and relevant EC, as appropriate.

The investigator may deviate from or change a protocol without prior ethics approval *ONLY* to eliminate an immediate hazard(s) to trial subjects. As soon as possible, the implemented deviation or change, the reasons for it, and, if appropriate, the proposed protocol amendment(s) should be submitted to:

A. the local Ethics Committee for review and approval and, if required,

B. the Sponsor for agreement

16 PLANS FOR DISTRIBUTION OF RESEARCH FINDINGS TO STUDY COMMUNITY

At the end of the study, a research report of the methods, detailed results, and brief conclusions will be prepared for distribution to the collaborators. A simplified lay document will be made available to study subjects. The results will be explained in lay terms to the local community at the Sukuta Open Day event which is held annually. This may be attended by local press, radio and television.

17 REFERENCES

1. Rousseau CM, Nduati RW, Richardson BA, Steele MS, John-Stewart GC, et al. (2003) Longitudinal analysis of human immunodeficiency virus type 1 RNA in breast milk and of its relationship to infant infection and maternal disease J Infect Dis 187: 741-747.
2. Mbori-Ngacha D, Nduati R, John G, Reilly M, Richardson B, et al. (2001) Morbidity and mortality in breastfed and formula-fed infants of HIV-1-infected women: A randomizedclinical trial. JAMA 286: 2413-2420.
3. Wherry EJ, Teichgraber V, Becker TC, Masopust D, Kaech SM, et al. (2003) Lineage relationship and protective immunity of memory CD8 T cell subsets Nat Immunol 4: 225-234.
4. Adkins B, Leclerc C, Marshall-Clarke S (2004) Neonatal adaptive immunity comes of age Nat Rev Immunol 4: 553-564.
5. Goulder PJ, Jeena P, Tudor-Williams G, Burchett S (2001) Paediatric HIV infection: correlates of protective immunity and global perspectives in prevention and management Br Med Bull 58: 89-108.
6. Marchant A, Newport M (2000) Prevention of infectious diseases by neonatal and early infantile immunization: prospects for the new millennium Curr Opin Infect Dis 13: 241-246.
7. Siegrist CA (2001) Neonatal and early life vaccinology Vaccine 19: 3331-3346.
8. Buseyne F, Burgard M, Teglas JP, Bui E, Rouzioux C, et al. (1998) Early HIV-specific cytotoxic T lymphocytes and disease progression in children born to HIV-infected mothers AIDS Res Hum Retroviruses 14: 1435-1444.
9. Luzuriaga K, Holmes D, Hereema A, Wong J, Panicali DL, et al. (1995) HIV-1-specific cytotoxic T lymphocyte responses in the first year of life J Immunol 154: 433-443.
10. Pikora CA, Sullivan JL, Panicali D, Luzuriaga K (1997) Early HIV-1 envelope-specific cytotoxic T lymphocyte responses in vertically infected infants J Exp Med 185: 1153-1161.
11. Marchant A, Appay V, Van Der Sande M, Dulphy N, Liesnard C, et al. (2003) Mature CD8(+) T lymphocyte response to viral infection during fetal life J Clin Invest 111: 1747-1755.
12. Hussey GD, Watkins ML, Goddard EA, Gottschalk S, Hughes EJ, et al. (2002) Neonatal mycobacterial specific cytotoxic T-lymphocyte and cytokine profiles in response to distinct BCG vaccination strategies Immunology 105: 314-324.
13. Marchant A, Goetghebuer T, Ota MO, Wolfe I, Ceesay SJ, et al. (1999)

Newborns develop a Th1-type immune response to Mycobacterium bovis bacillus Calmette-Guerin vaccination J Immunol 163: 2249-2255.

14. Ota MO, Vekemans J, Schlegel-Haueter SE, Fielding K, Sanneh M, et al. (2002) Influence of Mycobacterium bovis bacillus Calmette-Guerin on antibody and cytokine responses to human neonatal vaccination J Immunol 168: 919-925.
15. Vekemans J, Amedei A, Ota MO, D'Elios MM, Goetghebuer T, et al. (2001) Neonatal bacillus Calmette-Guerin vaccination induces adult-like IFN-gamma production by CD4+ T lymphocytes Eur J Immunol 31: 1531-1535.
16. Marchant A, Goldman M (2005) T cell-mediated immune responses in human newborns: ready to learn? Clin Exp Immunol 141: 10-18.
17. McShane H, Pathan AA, Sander CR, Keating SM, Gilbert SC, et al. (2004) Recombinant modified vaccinia virus Ankara expressing antigen 85A boosts BCG-primed and naturally acquired antimycobacterial immunity in humans Nat Med 10: 1240-1244.
18. Hanke T, McMichael AJ (2000) Design and construction of an experimental HIV-1 vaccine for a year-2000 clinical trial in Kenya. Nat Med 6: 951-955.
19. Slyker JA, Lohman BL, Mbori-Ngacha DA, Reilly M, Wee EG, et al. (2005) Modified vaccinia Ankara expressing HIVA antigen stimulates HIV-1-specific CD8 T cells in ELISpot assays of HIV-1 exposed infants Vaccine 23: 4711-4719.
20. Dorrell L, Yang H, Iversen AK, Conlon C, Suttill A, et al. (2005) Therapeutic immunization of highly active antiretroviral therapy-treated HIV-1-infected patients: safety and immunogenicity of an HIV-1 gag/poly-epitope DNA vaccine Aids 19: 1321-1323.
21. Dorrell L, Yang H, Ondondo B, Dong T, di Gleria K, et al. (2006) Expansion and diversification of HIV-1-specific T cells following immunisation of HIV-1-infected individuals with a recombinant modified vaccinia virus Ankara / HIV-1 gag vaccine. J Virol 80: 4705-4716.
22. Hanke T, Blanchard TJ, Schneider J, Ogg GS, Tan R, et al. (1998) Immunogenicities of intravenous and intramuscular administrations of MVA-based multi-CTL epitope vaccine for HIV in mice. J Gen Virol 79: 83-90.
23. Im EJ, Saubi N, Virgili G, Sander C, Teoh D, et al. (2007) Vaccine platform for prevention of tuberculosis and mother-to-child transmission of human immunodeficiency virus type 1 through breastfeeding J Virol 81: 9408-9418.
24. Letourneau S, Im E-J, Mashishi T, Brereton C, Bridgeman A, et al. (2007) Design and pre-clinical evaluation of a universal HIV-1 vaccine PLoS ONE.
25. Hanke T, Samuel RV, Blanchard TJ, Neumann VC, Allen TM, et al. (1999) Effective induction of simian immunodeficiency virus-specific cytotoxic T lymphocytes in macaques by using a multiepitope gene and DNA prime-modified vaccinia virus Ankara boost vaccination regimen J Virol 73: 7524-7532.
26. Im E-J, di Gleria K, McMichael AJ, Hanke T (2006) Induction of long-lasting multi-specific $CD8^+$ T cells by a 4-component DNA-MVA/HIVA-RENTA candidate HIV-1 vaccine in rhesus macaques Eur J Immunol 36: 2574-2584.
27. Robinson HL, Montefiori DC, Johnson RP, Manson KH, Kalish ML, et al. (1999) Neutralizing antibody-independent containment of immunodeficiency virus challenges by DNA priming and recombinant pox virus booster immunizations Nat Med 5: 526-534.
28. Seth A, Ourmanov I, Schmitz JE, Kuroda MJ, Lifton MA, et al. (2000)

Immunization with a modified vaccinia virus expressing simian immunodeficiency virus (SIV) Gag-Pol primes for an anamnestic Gag-specific cytotoxic T-lymphocyte response and is associated with reduction of viremia after SIV challenge. J Virol 74: 2502-2509.

29. Amara RR, Villinger F, Altman JD, Lydy SL, O'Neil SP, et al. (2001) Control of a Mucosal Challenge and Prevention of AIDS by a Multiprotein DNA/MVA Vaccine Science 292: 69-74.
30. Stittelaar KJ, Kuiken T, de Swart RL, van Amerongen G, Vos HW, et al. (2001) Safety of modified vaccinia virus Ankara (MVA) in immune-suppressed macaques Vaccine 19: 3700-3709.
31. Hanke T, McMichael AJ, Samuel RS, Powell LAJ, McLoughlin L, et al. (2002) Lack of toxicity and persistence in the mouse associated with administration of candidate DNA- and modified vaccinia virus Ankara (MVA)-based HIV vaccines for Kenya Vaccine 21: 109-115.
32. Hanke T, McMichael AJ, Dennise MJ, Sharpe SA, Powell LAJ, et al. (2005) Biodistribution and persistence of an MVA-vectored candidate HIV vaccine in SIV-infected rhesus macaques and SCID mice. Vaccine 23: 1507-1514.
33. Estcourt MJ, Letourneau S, McMichael AJ, Hanke T (2005) Vaccine route, dose and type of delivery vector determine patterns of primary CD8+ T cell responses Eur J Immunol 35: 2532-2540.
34. Estcourt MJ, McMichael AJ, Hanke T (2005) Altered primary CD8(+) T cell response to a modified virus Ankara(MVA)-vectored vaccine in the absence of CD4(+) T cell help Eur J Immunol.
35. Hanke T, Barnfield C, Wee EG-T, Ågren L, Samuel RV, et al. (2003) Construction and immunogenicity in a prime-boost regimen of a Semliki Forest virus-vectored experimental HIV clade A vaccine J Gen Virol 84: 361-368.
36. Larke N, Murphy A, Wirblich C, Teoh D, Estcourt MJ, et al. (2005) Induction of human immunodeficiency virus type 1-specific T cells by a bluetongue virus tubule-vectored vaccine prime-recombinant modified virus Ankara boost regimen J Virol 79: 14822-14833.
37. Nkolola JP, Wee EG-T, Im E-J, Jewell CP, Chen N, et al. (2004) Engineering RENTA, a DNA prime-MVA boost HIV vaccine tailored for Eastern and Central Africa Gene Ther 11: 1068-1080.
38. Nordstrom EK, Forsell MN, Barnfield C, Bonin E, Hanke T, et al. (2005) Enhanced immunogenicity using an alphavirus replicon DNA vaccine against human immunodeficiency virus type 1 J Gen Virol 86: 349-354.
39. Wee EG-T, Patel S, McMichael AJ, Hanke T (2002) A DNA/MVA-based candidate HIV vaccine for Kenya induces multi-specific T cell responses in rhesus macaques. J Gen Virol 83: 75-80.
40. Mayr A, Hochstein-Mintzel V, Stickl H (1975) Abstammung, Eigenschaften und Verwendung des attenuierten Vaccinia-Stammes MVA. Infection 105: 6-14.
41. Mayr A, Stickl H, Muller HK, Danner K, Singer H (1978) Der Pockenimpfstamm MVA: Marker, genetische Struktur, Erfahrungen mit der parenteralen Schutzimpfung und Verhalten im abwehrgeschwachten Organismus. Zentralbl Bakt Hyg I (Abt Orig B) 167: 375-390.
42. Bejon P, Peshu N, Gilbert SC, Lowe BS, Molyneux CS, et al. (2006) Safety profile of the viral vectors of attenuated fowlpox strain FP9 and modified vaccinia virus Ankara recombinant for either of 2 preerythrocytic malaria

antigens, ME-TRAP or the circumsporozoite protein, in children and adults in Kenya Clin Infect Dis 42: 1102-1110.

43. Cosma A, Nagaraj R, Buhler S, Hinkula J, Busch DH, et al. (2003) Therapeutic vaccination with MVA-HIV-1 nef elicits Nef-specific T-helper cell responses in chronically HIV-1 infected individuals Vaccine 22: 21-29.
44. Harrer E, Bauerle M, Ferstl B, Chaplin P, Petzold B, et al. (2005) Therapeutic vaccination of HIV-1-infected patients on HAART with a recombinant HIV-1 nef-expressing MVA: safety, immunogenicity and influence on viral load during treatment interruption Antivir Ther 10: 285-300.
45. Hanke T, McMichael AJ, Dorrell L (2007) Clinical experience with plasmid DNA- and modified vaccinia vaccine Ankara (MVA)-vectored HIV-1 clade A vaccine inducing T cells J Gen Virol 88: 1-12.
46. Cebere I, Dorrell L, McShane H, Simmons A, McCormack S, et al. (2006) Phase I clinical trial safety of DNA- and modified virus Ankara-vectored human immunodeficiency virus type 1 (HIV-1) vaccines administered alone and in a prime-boost regime to healthy HIV-1-uninfected volunteers Vaccine 24: 417-425.
47. Yang H, Dong T, Turnbull E, Ranasinghe S, Ondondo B, et al. (2007) Broad TCR usage in functional HIV-1-specific CD8+ T cell expansions driven by vaccination during highly active antiretroviral therapy J Immunol 179: 597-606.
48. Goonetilleke N, Moore S, Dally L, Winstone N, Mahmoud N, et al. (2006) Prime-boost vaccination with recombinant DNA and MVA expressing HIV-1 Clade A gag and immunodominant CTL epitopes induces multi-functional HIV-1-specific T cells in healthy subjects J Virol 80: 4717-4728.

APPENDIX 1 - EQUIPMENT FOR FIELDWORK

Field Team
Field workers / SENs
Field worker supervisor
Study physician

Transport
Landrover x 1 with driver
Motorbikes for field workers for home visits

Equipment
Bags to carry equipment
Address of subjects
Mobile phones
Pens, note books
Study forms / CRFs
Cold boxes
Dry ice
Sharpsafe box
Alcohol swabs, cotton swabs, tissue paper
Scales for weighing, tape measure
Height measuring equipment
MVA.HIVA vials
EPI vaccines
Syringes and needles
Test tubes for blood samples
Drugs (analgesics, antimalarials, antibiotics), antihistamines, multivitamins
Resuscitation box containing Ambu bag and face masks, endotracheal tubes, oropharyngeal airways, laryngoscopes and blades, batteries
Resuscitation drugs (adrenaline, antihistamines, hydrocortisone, intravenous fluids, oxygen)
